# A comprehensive SARS-CoV-2-human protein-protein interactome network identifies pathobiology and host-targeting therapies for COVID-19

**DOI:** 10.21203/rs.3.rs-1354127/v2

**Published:** 2022-06-07

**Authors:** Yadi Zhou, Yuan Liu, Shagun Gupta, Mauricio I. Paramo, Yuan Hou, Chengsheng Mao, Yuan Luo, Julius Judd, Shayne Wierbowski, Marta Bertolotti, Mriganka Nerkar, Lara Jehi, Nir Drayman, Vlad Nicolaescu, Haley Gula, Savaş Tay, Glenn Randall, John T. Lis, Cédric Feschotte, Serpil C. Erzurum, Feixiong Cheng, Haiyuan Yu

**Affiliations:** 1 Genomic Medicine Institute, Lerner Research Institute, Cleveland Clinic, Cleveland, OH 44195, US; 2 Weill Institute for Cell and Molecular Biology, Cornell University, Ithaca, NY 14853, US; 3 Department of Computational Biology, Cornell University, Ithaca, NY 14853, US; 4 Department of Molecular Biology and Genetics, Cornell University, Ithaca, NY 14853, US; 5 Division of Health and Biomedical Informatics, Department of Preventive Medicine, Northwestern University, Chicago, IL 60611, US; 6 Lerner Research Institute, Cleveland Clinic, Cleveland, OH 44195, US; 7 Pritzker School of Molecular Engineering, The University of Chicago, Chicago, IL 60637, US; 8 Department of Microbiology, Ricketts Laboratory, University of Chicago, Chicago, IL 60637, US; 9 Case Comprehensive Cancer Center, Case Western Reserve University School of Medicine, Cleveland, OH 44106, US; 10 Department of Molecular Medicine, Cleveland Clinic Lerner College of Medicine, Case Western Reserve University, Cleveland, OH 44195, US

**Keywords:** Interactomics, SARS-CoV-2

## Abstract

Physical interactions between viral and host proteins are responsible for almost all aspects of the viral life cycle and the host’s immune response. Studying viral-host protein-protein interactions is thus crucial for identifying strategies for treatment and prevention of viral infection. Here, we use high-throughput yeast two-hybrid and affinity purification followed by mass spectrometry to generate a comprehensive SARS-CoV-2-human protein-protein interactome network consisting of both binary and co-complex interactions. We report a total of 739 high-confidence interactions, showing the highest overlap of interaction partners among published datasets as well as the highest overlap with genes differentially expressed in samples (such as upper airway and bronchial epithelial cells) from patients with SARS-CoV-2 infection. Showcasing the utility of our network, we describe a novel interaction between the viral accessory protein ORF3a and the host zinc finger transcription factor ZNF579 to illustrate a SARS-CoV-2 factor mediating a direct impact on host transcription. Leveraging our interactome, we performed network-based drug screens for over 2,900 FDA-approved/investigational drugs and obtained a curated list of 23 drugs that had significant network proximities to SARS-CoV-2 host factors, one of which, carvedilol, showed promising antiviral properties. We performed electronic health record-based validation using two independent large-scale, longitudinal COVID-19 patient databases and found that carvedilol usage was associated with a significantly lowered probability (17%−20%, *P* < 0.001) of obtaining a SARS-CoV-2 positive test after adjusting various confounding factors. Carvedilol additionally showed anti-viral activity against SARS-CoV-2 in a human lung epithelial cell line [half maximal effective concentration (EC_50_) value of 4.1 μM], suggesting a mechanism for its beneficial effect in COVID-19. Our study demonstrates the value of large-scale network systems biology approaches for extracting biological insight from complex biological processes.

The global coronavirus disease 2019 (COVID-19) pandemic caused by the highly transmissible and pathogenic severe acute respiratory syndrome coronavirus 2 (SARS-CoV-2) remains a persistent part of everyday life for much of the world. Epidemiological models predict, in line with virological theory, that the pandemic is unlikely to end with the complete eradication of the virus^1,2^. Indeed, SARS-CoV-2 becoming endemic in pockets of the world’s population is hypothesized to be a natural consequence of the virus’s widespread propagation^2^, and the emergence of numerous viral variants raise concern of a perennial selection for more infectious or virulent mutants to again sweep through the globe. This highlights the need to fill in the gaps in our understanding of the interplay between this virus and its host upon natural infection and immunization, and thus, much work remains to be done to elucidate the pathobiology of SARS-CoV-2, especially as the maintenance of immunity against this pathogen remains at utmost interest to global public health.

Viruses interface with host cell surfaces to gain entry, wherein they interact with intracellular proteins to hijack host mechanisms that facilitate viral replication and evasion of an immune response^3^. Studying viral-host protein-protein interactions (PPIs) is therefore pivotal for understanding the mechanisms by which viral infection progresses, how the host responds to said infection, and thus, for identifying strategies for treatment and prevention^4–7^. These networks of interactions are especially important as proteins rarely act in isolation and their roles should be evaluated in conjunction with their neighborhood of interacting partners. Such interactomes can thus reveal biological pathways and processes impacted by the viral proteome, allowing for the discovery of novel drug targets that directly or indirectly affect the viral-host point of contact.

To that effect, here we leverage high-throughput yeast two-hybrid (Y2H) and tandem mass tag affinity purification followed by mass spectrometry (TMT-AP/MS) to generate the first binary and co-complex SARS-CoV-2-human protein-protein interactome network, which we propose to be a more complete resource for exploration of the viral-host interactome (Fig. 1a). We adopted this approach for several reasons. To date, Y2H and AP/MS are the only two methodologies available for mapping protein-protein interactome networks on a proteomic scale^8,9^. Pioneering studies publishing the earliest SARS-CoV-2-human interactomes utilized label-free AP/MS as their sole method for interaction mapping^4–7^. While both Y2H and AP/MS alone produce high-quality interactome datasets, they fundamentally capture different yet complementary aspects of the full network; specifically, Y2H interactions represent key connections between different protein complexes and pathways^10^. Thus, Y2H and AP/MS together can provide a more comprehensive view of the topological and biological properties of the interactome^10^. Moreover, labelled (e.g., TMT-based) AP/MS has been shown to provide more precise, accurate, and reproducible quantification of proteins compared to label-free AP/MS-based approaches, which is an important criterion when trying to identify true protein interactions and generate high-quality interactome networks^11–21^.

Here, we used both Y2H and quantitative TMT-AP/MS to generate a total of 739 high-confidence interactions among 579 human proteins and 28 SARS-CoV-2 proteins. Our interactome had an unprecedented scale and coverage compared with existing ones. Using our interactome, we identified important pathways such as protein translation, mRNA splicing, Golgi transportation, neutrophil mediated immunity, and glucose metabolism. Moreover, we prioritized host-targeting therapies by searching U.S. FDA-approved/investigational drugs for their potential anti-SARS-CoV-2 effect using state-of-the-art network proximity methods. Using two large independent COVID-19 patient databases, we found that usage of one of the top candidates, carvedilol, was associated with a lowered risk (17%−20%) of a positive COVID-19 test. Experimental validation shows that carvedilol inhibits SARS-CoV-2 infection with a half maximal effective concentration (EC_50_) of 4.1 μM. Altogether, these results suggest that our comprehensive SARS-CoV-2-human protein interactome offers great opportunities for understanding the pathobiological process of SARS-CoV-2 in human and identifying host-targeting therapies for COVID-19.

## Results

### A comprehensive SARS-CoV-2-human protein-protein interactome network

**Figure 1.**
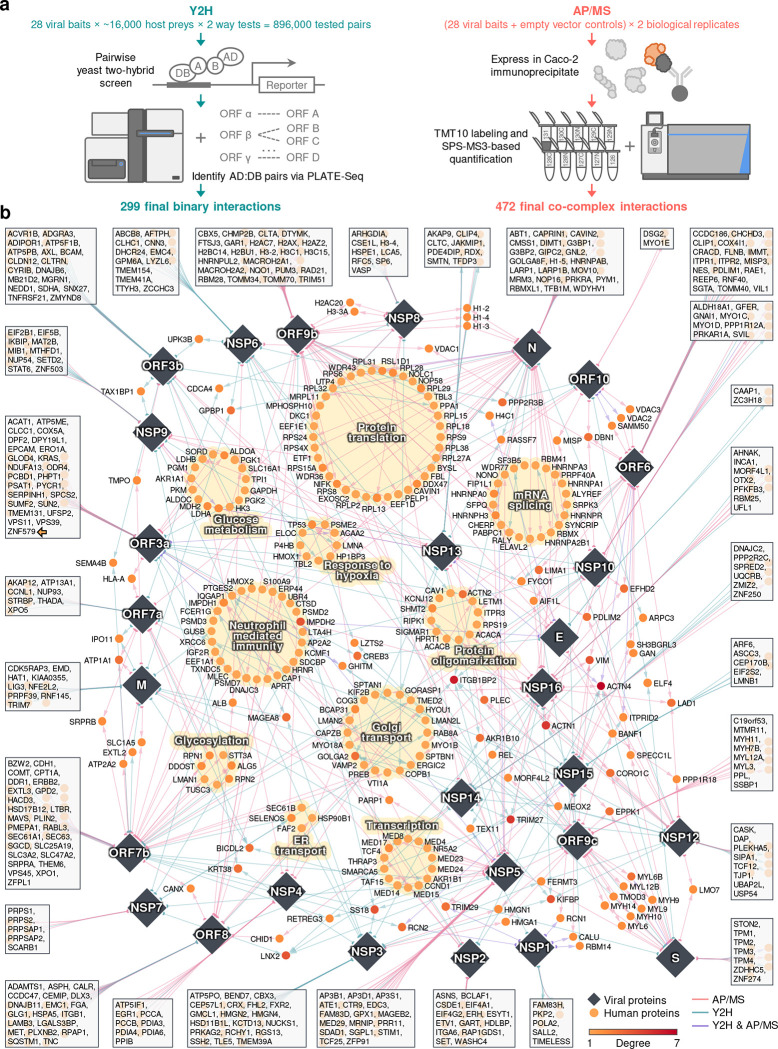
SARS-CoV-2-human protein interactome. (**a**) Pipelines using Y2H and AP/MS for detecting SARS-CoV-2-human protein-protein interactions. (**b**) Edges between viral proteins (diamonds) and human proteins (circles) represent protein-protein interactions. Edge colors indicate the methods used to detect the protein-protein interaction. Several biological processes that are significantly enriched in these human proteins (Fig. S2 and Table S2) are highlighted with yellow background. Human proteins that interact with only one SARS-CoV-2 protein are shown in the box connected to that specific protein. The interactome can be found in Table S1.

To generate a binary SARS-CoV-2-human protein-protein interactome, we systematically tested all pairwise combinations of 28 SARS-CoV-2 proteins (GenBank entry - MN908947) against ~16,000 human proteins (hORFeome V8.1)^22^ using high-throughput Y2H screens^10,23–26^ (Fig. 1a). We treated each protein as both a bait and a prey, yielding over 896,000 (28 × ~16,000 × 2) total tested pair combinations. Prior to screening, all autoactivating DNA-binding domain (DB) ORF clones were removed from further tests (see **Methods**). To increase experimental throughput, viral ORF activating domain (AD) and DB clones were mated against pools of 24 human ORF DB or AD clones, respectively. Following auxotrophic selection, AD:DB pairs were identified via PLATE-seq^26^ to generate a list of candidate interactions (see **Methods**). Interaction candidates were then subsequently re-tested using Y2H to ascertain high reproducibility. In all, we report a total of 299 high-quality binary SARS-CoV-2-human PPIs via our high-throughput Y2H screen, 267 of which were unique to this assay in this study (Table S1).

To complement our binary SARS-CoV-2-human protein-protein interactome, we independently expressed each of the 28 SARS-CoV-2 proteins in the human intestinal epithelial cell line Caco-2 (HTB-37; ATCC) to identify viral-host co-complex interactions using TMT-AP/MS proteomics (Fig. 1a). We used Caco-2 as our cell line model due to its endogenous expression of angiotensin-converting enzyme 2 (ACE2) and transmembrane serine protease 2 (TMPRSS2) required for SARS-CoV-2 cell entry and S protein priming, respectively^27^, the line’s extensive use in SARS-CoV and SARS-CoV-2 infection studies^28,29^, supported by known *in vivo* replication of SARS-CoV-2 in gastrointestinal cells^30,31^, and desirable cell culture characteristics including robust transfectability and rapid propagation. All Strep-, Myc-, or FLAG-tagged SARS-CoV-2 baits and their corresponding empty vector controls were transfected in biological duplicates, followed by subsequent affinity purification, TMT10 labeling, and SPS-MS3-based quantification. We filtered for interactions that met stringent fold change and *p*-value based cutoffs (see **Methods**). In all, we report a total of 472 high-confidence co-complex SARS-CoV-2-human PPIs via AP/MS, 440 of which were unique to this assay in this study (Table S1). Altogether, our orthogonal approaches generated a network composed of 739 interactions among 28 viral and 579 host proteins (Table S1).

We visualized the SARS-CoV-2-human protein-protein interactome through a network shown in Fig. 1b. The colors of the edges between the viral proteins (represented as diamond nodes) and the host proteins (represented as circle nodes) indicate the methods that detected the interaction. Host proteins that interact with a single viral protein are shown in boxes connected to their interacting partner. Several human proteins interact with multiple SARS-CoV-2 proteins, such as ACTN4, ITGB1BP2, TRIM27, and ACTN1, while the majority of human proteins (469, 81%) interact with only one SARS-CoV-2 protein (Fig. S1a). Among the viral proteins, N, ORF7b, and ORF9b achieved the highest network degrees, whereas E, NSP7, and NSP1 have the lowest network degrees (Fig. S1b). In terms of the shared interacting partners, overall, the viral proteins showed low overlap (Fig. S1c), consistent with a previously published SARS-CoV-2 interactome network^4,5^. We examined the overlap of host factors for Y2H and AP/MS separately and found overall low overlap of host factors as well (Fig. S1d–e).

For the entire interactome, functional enrichment analysis revealed significantly overrepresented biological processes (Fig. S2a and Table S2), including protein translation, transcription, and neutrophil-mediated immunity (highlighted with yellow background in Fig. 1b). Sematic analysis shows major biological process categories such as “ribosome biogenesis,” “rRNA metabolic process,” and “viral gene expression” (Fig. S2b). Pathway enrichment analysis show top enriched pathways such as “protein processing in the endoplasmic reticulum,” “tight junction,” “glycolysis,” “ribosome,” and “protein export” (Fig. S2c and Table S2). For individual SARS-CoV-2 proteins, many pathways and biological processes are shared in these viral proteins (Fig. S3). For example, NSP12, NSP13, and NSP16 share biological processes such as “regulation of cellular component movement,” “negative regulation of cell morphogenesis involved in differentiation,” and “negative regulation of substrate adhesion-dependent cell spreading” (Fig. S3a), and ORF7a, ORF7b, ORF8, and NSP4 share the pathway “protein processing in endoplasmic reticulum” (Fig. S3b).

Overall, our interactome is comprised of abundant information that can be utilized for the identification of COVID-19-relevant pathobiology and host-targeting therapies. We also developed an interactive visualization tool for our interactome which can be accessed from https://github.com/ChengF-Lab/COVID-19_PPI.

### This SARS-CoV-2-human interactome is of high coverage and quality

**Extended Figure 1.**
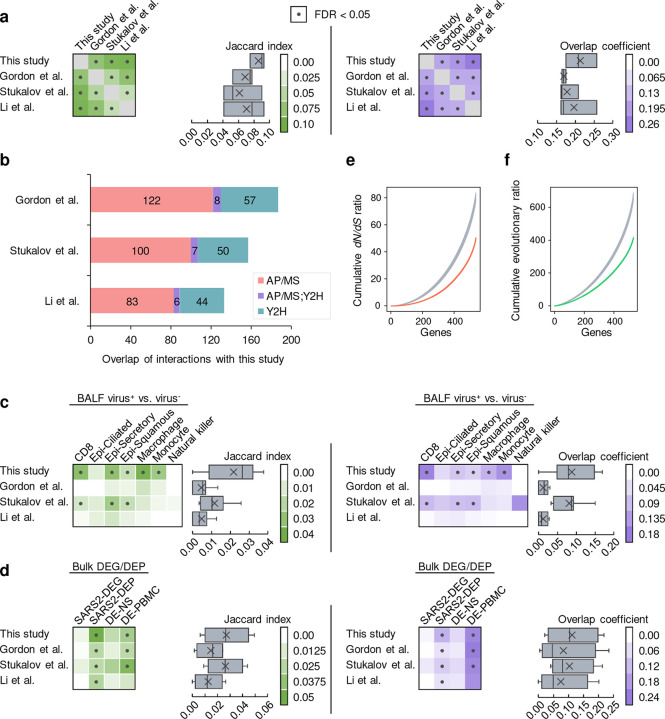
Characteristics of the interactome. (**a**) Overlap of the host factors among the four interactomes compared in this study. Heatmaps show the Jaccard indexes (green) and overlap coefficients (purple) of the host factors against other gene sets. Dots indicate FDR < 0.05 by Fisher’s exact test. In the box plots, boxes range from lower to upper quartiles, center lines indicate medians, whiskers show 1.5 × interquartile ranges, and crosses show mean values. (**b**) The overlap of the interactions in our interactome with the other three interactomes by considering the protein complexes and pathways. If two host factors interacting with the same viral protein are known to interact with each other in the literature, we consider the two viral-host interactions as overlapping. (**c**) Overlap of the host factors with the differentially expressed genes in SARS-CoV-2^+^ vs. SARS-CoV-2^−^ cells in seven cell types from COVID-19 patient samples. Epi - epithelial. (**d**) Overlap of the host factors with the differentially expressed genes from four bulk RNA-seq/proteomics datasets. (**e**, **f**) Biological characteristics of the SARS-CoV-2 host factors. The host factors have lower non-synonymous to synonymous substitutions (*dN/dS*) ratios (**e**) and lower evolutionary ratios (**f**) compared to random background (grey, mean ± standard deviation of 100 repeats using genes randomly selected by degree preserved node shuffling). Genes were sorted in ascending order in terms of *dN/dS* ratio or evolutionary ratio.

**Figure 2.**
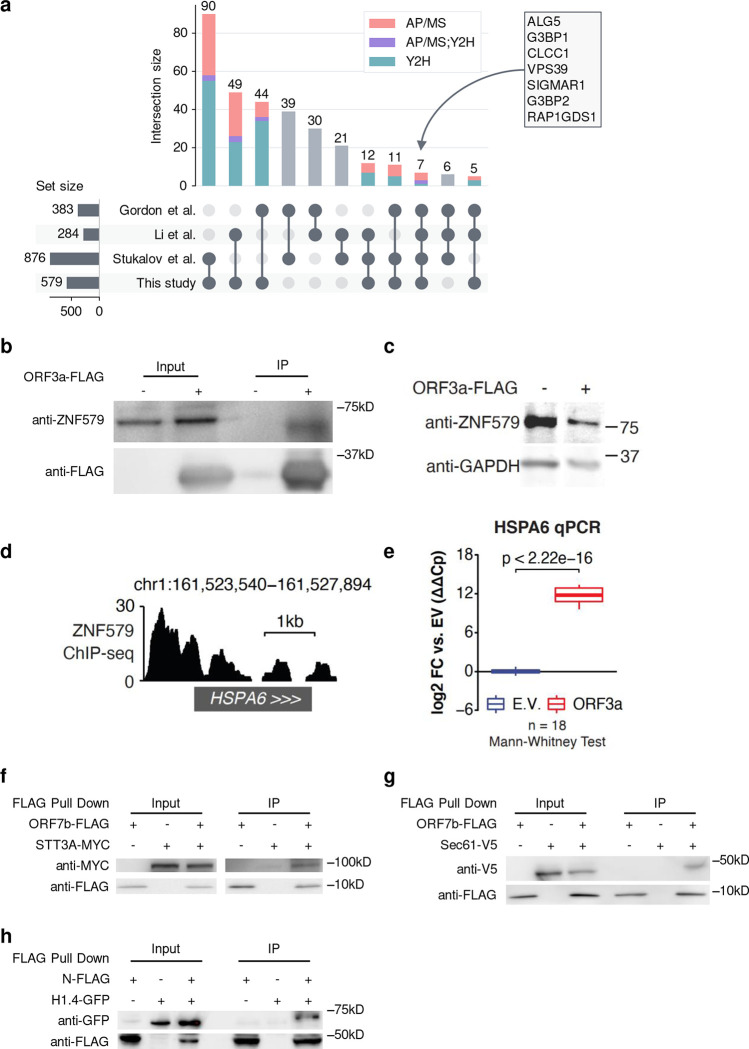
Characteristics of the interactome and validation of novel SARS-CoV-2-human interactions. (**a**) UpSet plot showing the overlap of SARS-CoV-2-human protein-protein interactions from four studies (Table S3). Each bar shows the interactions shared by only the marked studies at the bottom. Composition of each bar in terms of the source of the interactions are indicated by different colors. **(b)** Co-immunoprecipitation confirming ORF3a-ZNF579 interaction in HEK 293T cells following transfection with ORF3a-FLAG or empty vector. **(c)** Western blot showing levels of ZNF579 along with GAPDH as a loading control in 293T cells following transfection with ORF3a-FLAG or empty vector. **(d)** ChIP-seq for ZNF579 in MCF7 cells from the ENCODE consortium at the *HSPA6* locus. Signal is log2 fold change over input. (**e**) Expression of HSPA6 after transfection with ORF3a-FLAG or empty vector. Two transfection replicates were probed with two primer pairs to HSPA6 at three different template dilutions in technical triplicate (18 total reactions for each condition). Expression is normalized to GAPDH and then to the empty vector average using the double-delta Ct method. (**f**-**g**) Co-immunoprecipitation confirming ORF7b-STT3A and ORF7b-Sec61 interactions in HEK293T cells following transfection with ORF7b-FLAG or empty vector and STT3A-MYC or Sec61-V5, respectively. (**h**) Co-immunoprecipitation confirming N-histone H1.4 interaction in HEK293T cells following transfection with N or empty vector and histone H1.4.

To ensure the authenticity when applying our interactome for downstream studies, we first evaluated the quality through several means. We examined three previously published SARS-CoV-2-human protein-protein interactome networks^4,6,7^. Importantly, all three of these interactomes were generated using AP/MS-based methods alone. Overall, we found that the host factors of these interactomes significantly overlap (Fisher’s exact test, FDR < 0.05) (Fig. 2a, Extended Fig. 1a, and Table S3), although each interactome still identified a large number of unique factors. This could be explained by differences in the cell line models used (Gordon et al. and Li et al. used HEK-293T/17; Stukalov et al. used A549) as well as distinct computational and/or experimental methodologies implemented in their respective study. Still, we found that our interactome had the highest overlap of interaction partners among published SARS-CoV-2-human protein-protein interactome networks (Fig. 2a, Extended Fig. 1a, and Table S3), suggesting that our interactome had a high level of coverage. We also found that Y2H resulted in slightly more overlapping host factors (Fig. 2a), confirming the complementary nature of Y2H interactions, which are independent of human cells. Overall, AP/MS achieved significantly more overlapping viral-human protein-protein interactions across studies when known human pathways and complexes are taken into consideration (Extended Fig. 1b).

Compared to the published SARS-CoV-2-human protein-protein interactome networks^4,6,7^, our interactome validated 218 (38%) human host factors previously reported. Proteins including ALG5, G3BP1, CLCC1, VPS39, SIGMAR1, G3BP2, and RAP1GDS1 are identified in all four interactomes (Fig. 2a). Importantly, our interactome offers 361 (62%) newly discovered human host factors which in total interact with SARS-CoV-2 proteins in 493 novel interactions. For S protein, which plays a key role in the entry of SARS-CoV-2 into host cells^32^, we identified 24 novel interacting partners. Among these interacting partners of S protein, we found that CORO1C^33^ and STON2^34^ express on the cell membrane, suggesting potential cell entry of SARS-CoV-2 through these human proteins in addition to known mechanisms.

We next performed several comparisons using these interactomes and our own. First, we found that our interactome was enriched in several disease-relevant pathways and biological processes that were not enriched in previous interactomes, for example, “Coronavirus disease, ” “ribosome biogenesis,” and “rRNA metabolic process”^35–38^ (Table S2). Next, we examined whether these datasets contained interaction partners that coincided with genes that had expression changes in response to SARS-CoV-2 infection. To this end, we performed differential expression analysis for several bulk and single-cell RNA-seq datasets from COVID-19 models or patients (see **Methods**). For the single-cell dataset^39^ which we compared the gene expression in SARS-CoV-2^+^ and SARS-CoV-2^−^ cells, we found that our interactome showed significant overlap (Fisher’s exact test, FDR < 0.05) with the differentially expressed genes (DEGs) in more cell types than that of other interactomes (Extended Fig. 1c and Table S3). Using four bulk RNA-seq/proteomics datasets that contained samples such as upper airway and bronchial epithelial cells^40–43^, we found that our interactome had a comparable number of significant overlaps to other datasets and showed the highest overall Jaccard index and overlap coefficient with the bulk RNA-seq datasets (Extended Fig. 1d and Table S3). These results suggest that our interactome is highly enriched in genes differentially expressed in response to SARS-CoV-2 infection.

We next inspected the evolutionary features of the SARS-CoV-2 human host factors (Extended Fig. 1e–f and Table S4). Previous studies have shown that virus host factors have more conserved dN/dS rates compared to non-virus host factors^44,45^. Our SARS-CoV-2-human interactome showed more purifying selection (quantified by lower non-synonymous versus synonymous substitution rate ratio [*dN/dS* ratio]), as well as a lower evolutionary rate ratio, compared to the random background from the human protein interactome. These bioinformatics observations further suggested high evolutionary conservation of host factors of SARS-CoV-2 identified by our Y2H and TMT-AP/MS proteomics platforms, consistent with previous studies^46,47^.

Gene expression patterns in disease-related tissues capture important information for revealing the pathogenesis of the disease and identifying potential treatments^48–50^. We therefore examined the expression of the human host factors in different tissues (Fig. S4a–b and Table S5) using the GTEx data^51^. By normalizing the expression of each gene across different tissues (tissue specificity, see **Methods**), we found that lung ranked the 7^th^ out of 33 tissues in terms of the number of host factors with positive tissue specificity (Fig. S4a), suggesting that lung is one of the tissues where these host factors have high expression^52^.

Altogether, these results show the high quality of the SARS-CoV-2-human interactome identified in this study and strongly encouraged us to further look into the pathobiology of COVID-19 and potential treatment using our interactome.

### This SARS-CoV-2-human interactome has the potential to identify pathobiology of COVID-19

**Extended Figure 2.**
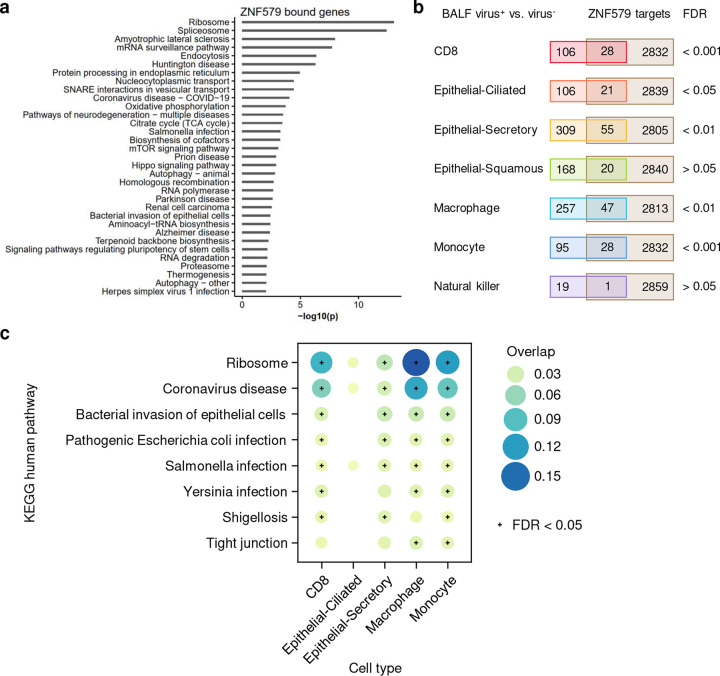
ZNF579 targets significantly overlap with the differentially expressed genes in SARS-CoV-2 infected patient samples. (**a**) Enriched KEGG pathways of genes associated with ZNF579 binding by ChIP-seq (ENCODE:ENCSR018MQH). Genes were considered to be bound by ZNF579 if a ChIP-seq peak overlapped with the promoter region (−1000 to transcription start site). (**b**) Overlap of ZNF579 targets and differentially expressed genes (DEGs) in bronchoalveolar lavage fluid (BALF) SARS-CoV-2^+^ vs. SARS-CoV-2^−^ samples. See **Methods** for the source of the single-cell dataset. Fisher’s exact tests show that the overlaps are significant (FDR < 0.05) for five cell types, including CD8, epithelial-ciliated, epithelial-secretory, macrophage, and monocyte. (**c**) The enriched pathways of the overlapping ZNF579 targets and DEGs in the five cell types. Pathways that are significantly enriched in at least two cell types are shown.

ORF3a is a SARS-CoV-2 accessory protein that has been reported to induce apoptosis in 293T cells^53^ and to suppress the innate immune response^54–56^ via unclear molecular mechanisms. Our interactome revealed that ORF3a physically interacts with ZNF579 (Fig. 1b, indicated by an arrow in the box of targets of ORF3a), a previously uncharacterized human protein likely to be a transcription factor. We were able to validate this interaction using co-immunoprecipitation (co-IP) western blotting (Fig. 2b). Furthermore, we found that the level of ZNF579 protein is decreased after overexpression of ORF3a in 293T cells (Fig. 2c). As a result, we hypothesized that the presence of ORF3a in cells might trigger changes in the transcriptional state of human genes that are normally regulated by ZNF579. Using ENCODE ChIP-seq data^57,58^, we found that there is a significant enrichment of genes known to be dysregulated in SARS-CoV-2 infection among targets bound by ZNF579 (Extended Fig. 2a–b). These overlapped genes participate in several disease-relevant pathways such as “ribosome,”^35–38^ “coronavirus disease,” and various infection-related pathways in several COVID-19 relevant cell types (Extended Fig. 2c). Specifically, ZNF579 is bound strongly to the promoter of *HSPA6* (Fig. 2d). Using qPCR, we found that overexpression of ORF3a in 293T cells causes massive induction of *HSPA6* (Fig. 2e). These results indicate that the multifunctional SARS-CoV-2 accessory protein ORF3a can induce expression of *HSPA6*, presumably by disrupting ZNF579, which is likely to normally exert repressive activity at the *HSPA6* promoter. This represents an additional previously unknown activity of this multifunctional viral accessory protein.

The oligosaccharyltransferase (OST) complex catalyzes the N-glycosylation of nascent polypeptides in the endoplasmic reticulum^59^. Glycoproteins are critical for normal cell-cell interactions, RNA replication and pathogenesis^60–62^. Interestingly, OST inhibitor has been shown to have activity against Dengue virus, Zika virus, West Nile virus, yellow fever viruses, and HSV1^63–65^ by affecting the viral replication. The OST complex was also found to be crucial for innate immune responses triggered by lipopolysaccharide^66^. Notably, the OST complex subunits STT3A/B, RPN1/2, and DDOST^67^ were all shown to be present in our Y2H and AP/MS interactome datasets, which we further validated using co-IP (Fig. 2f). Additionally, we also found Sec61 (Fig. 2g), which is a major component of the ER translocon that facilitates the entry of nascent polypeptides into the ER lumen for protein processing^68^. Evidence suggests that Sec61 may participate in the replication and transcription of several viruses like Ebola virus, Influenza virus, HIV, and Dengue virus^68–70^. Thus, we hypothesize that OST and Sec61 may also participate in SARS-COV-2 replication and/or the host immune response, offering potential targets for host-targeting therapy development.

The SARS-CoV-2 nucleocapsid (N) protein binds to the viral RNA genome and is multifunctional in viral RNA transcription, replication, and genome condensation^71–73^. N protein is conserved and stable with ~90% amino acid homology to the SARS-COV N protein^74^. From our dataset, we confirmed known interactions, including the stress granule core protein G3BP1/2 also found in three other interactome datasets. In addition to these known interactions, we identified a novel interaction between histone H1.4 and N protein. To validate this histone H1.4 and N protein interaction, we overexpressed both N protein and histone H1.4 to perform co-IP, confirming their interaction (Fig. 2h). Histone H1, also known as linker histone, mainly functions in chromatin condensation and transcriptional repression^75,76^. Accumulating evidence suggests that linker histone is essential in the pathogenesis of several diseases, particularly for viral infection^76^. There is also evidence that Histone H1 could regulate IFN and inhibit influenza replication^77^, in addition to playing a role in the regulation of viral gene expression^78^. Thus, we hypothesize that this novel viral-host interaction could also be involved in mediation viral replication and/or gene expression.

### Discovery of interactome-based host-targeting therapies for COVID-19

**Figure 3.**
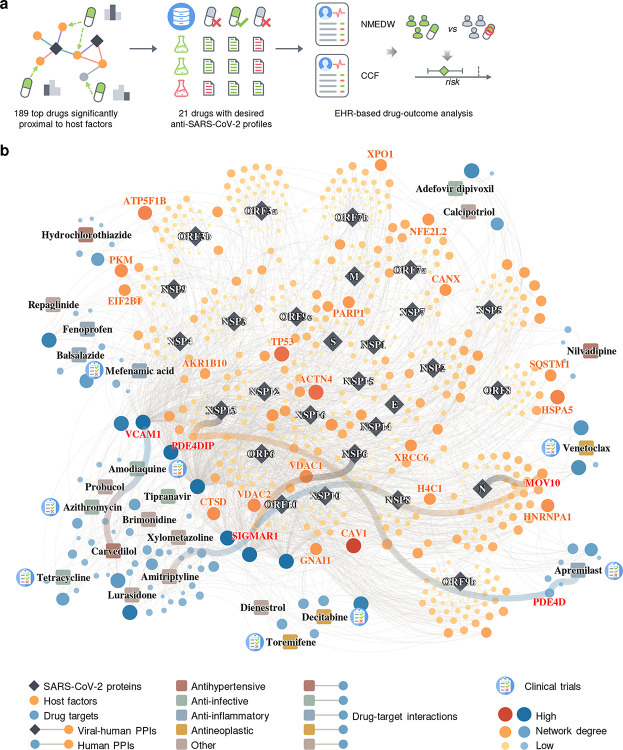
Discovery of interactome-based host-targeting therapies for COVID-19. (**a**) Workflow of drug repurposing for COVID-19 using our interactome. We ranked the drugs by their proximity to the SARS-CoV-2 host factors (Table S7), filtered the top drugs by their NCATS anti-SARS-CoV-2 profiles (Table S8), and finally analyzed their drug-outcome relationship using electronic health records (EHR) data (Table 1, Table S9–**S10**). (**b**) The top 23 drugs can target the SARS-CoV-2 host factors directly or through protein-protein interactions with their targets.

Using our newly discovered SARS-CoV-2-human protein-protein interactome network, we performed network-based drug screening for more than 2,900 FDA-approved/investigational drugs (Fig. 3a and Table S6)^79^. We obtained a list of 189 FDA-approved drugs with significantly closer network proximities to the SARS-CoV-2 host factors, among which 44 had clinical trials for SARS-CoV-2 (Table S7). To refine this list, we obtained the antiviral profiles of the top 189 drugs from NCATS (https://opendata.ncats.nih.gov/covid19/assays, National Center for Advancing Translational Sciences) and evaluated each drug for their desired antiviral properties (see **Methods**). From this, we obtained a curated list of 23 drugs with significant network proximities to the SARS-CoV-2 host factors as well as desired anti-SARS-CoV-2 activities in at least two NCATS assays (Fig. 3b, Fig. S5, and Table S8).

Overall, these top drugs fall into several major categories, including anti-infective (amodiaquine, azithromycin, tetracycline, adefovir dipivoxil, tipranavir), anti-inflammatory (apremilast, mefenamic acid, balsalazide, fenoprofen), antihypertensive (carvedilol, hydrochlorothiazide, nilvadipine), and antineoplastic (toremifene, decitabine, venetoclax). Among these drugs, apremilast, toremifene, decitabine, amodiaquine, and azithromycin are currently being or have been tested in clinical trials for SARS-CoV-2. These top 23 drugs offer candidate treatments for SARS-CoV-2 infections across diverse mechanism-of-actions identified from our human-SARS-CoV-2 interactome. For example, balsalazide, toremifene, tetracycline, venetoclax, tipranavir, and brimonidine may inhibit viral replication by inhibiting papain-like protease 3CL (Fig. S5 and Table S8). Other drugs, such as carvedilol and hydrochlorothiazide, may directly inhibit viral entry by disrupting the Spike-ACE2 PPI (Fig. S5 and Table S8). We also found some literature evidence that may provide mechanistic insights for these drugs against SARS-CoV-2 (highlighted in Fig. 3b). For example, apremilast is a phosphodiesterase 4D (PDE4D) inhibitor^80^, which interacts with PDE4DIP^81^, a direct target of NSP13. Amitriptyline activates SIGMAR1^82^, while NSP6 interact with SIGMAR1 to inhibit host autophagosome formation to facility coronavirus replication^83^. SIGMAR1 also interact with MOV10 (an RNA helicase, also a host factor targeted by the N protein) which exhibits antiviral activity against RNA viruses^84^.

**Extended Figure 3.**
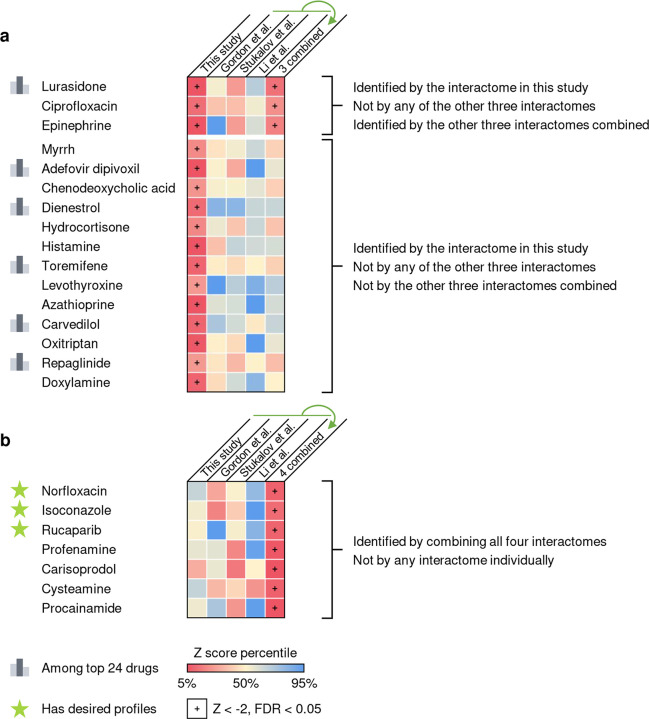
Comparison of the drug screening results using different interactomes and their combinations. (**a**) 16 drugs identified by our interactome cannot be identified by any of the other three interactomes (and the interactome combined from them for 13 drugs) compared in this study. 6 of the top 23 drugs with desired anti-SARS-CoV-2 profiles are among these drugs. (**b**) Drugs identified by combining all four interactomes that could not be identified by any interactome individually. Three drugs (highlighted with a star) were found to have desired anti-SARS-CoV-2 profiles.

To test whether our interactome identified drugs that could not be predicted by previously published datasets, we compared the screening results using different interactomes and their combinations. We found that 16 drugs identified by our interactome could not be predicted by any of the other three interactomes or their combinations (Extended Fig. 3a). Of the top 23 drugs with desired anti-SARS-CoV-2 profiles, six were among the 16 drugs that can only be identified by our interactome (Extended Fig. 3a). We also found that among the seven drugs identified by combining all four interactomes that could not be identified by any interactome individually, three drugs (Extended Fig. 3b, highlighted with a star) were found to have desired anti-SARS-CoV-2 profiles (Fig. S6).

Among the top 23 drug candidates, toremifene achieved significantly closer network proximity (Z = −2.19, FDR = 0.037) and has a desired anti-SARS-CoV-2 profile. Previous studies show that toremifene blocks various viral infections efficiently, including SARS-CoV-2^85^ [half maximal inhibitory concentration (IC_50_) = 3.58 μM], SARS-CoV-1^86^ (EC_50_ = 11.97 μM), MERS-CoV^87^ (EC_50_ = 12.9 μM), and Ebola virus^88^ (IC_50_ = ~1 μM). Indeed, NCATS data show that toremifene is active across four assays: Spike-ACE2 protein-protein interaction [half maximal activity concentration (AC_50_) = 11.92 μM], SARS-CoV pseudotyped particle entry (AC_50_ = 15.85 μM), MERS-CoV pseudotyped particle entry (AC_50_ = 31.62 μM), and 3CL enzymatic activity (AC_50_ = 5.01 μM) (Fig. S5). Mechanistically, a previous study that toremifene may inhibit SARS-CoV-2 cell entry by blocking the S and NSP14 proteins^89^. These comprehensive validations show potential implications of SARS-CoV-2 interactome-predicted drugs (e.g., toremifene) offer candidates to be tested further in COVID-19 patients.

### Population-based and experimental validation of interactome-predicted drugs

Further, we used subject matter expertise to select candidate drugs for patient-level data validation and experimental validation based on a combination of factors: (1) strength of the interactome network-based prediction associations (a stronger network proximity score in Table S7); (2) novelty of predicted drugs; (3) availability of sufficient patient data for meaningful evaluation (exclusion of infrequently used medications); and (4) ideal pharmacokinetics properties in lung of interactome-predicted drugs. Applying these criteria resulted in 2 top candidate drugs, carvedilol (*Z* = −2.195, *FDR* = 0.03) and hydrochlorothiazide (*Z* = −2.428, *FDR* = 0.005), which are originally approved for treatment of hypertension.

**Figure 4.**
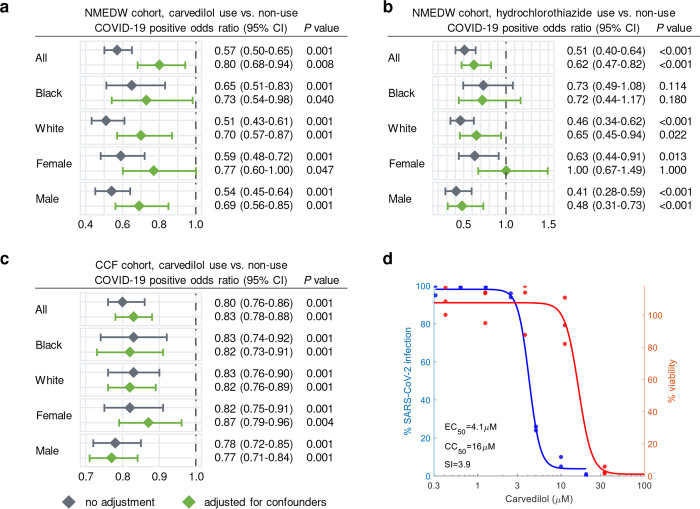
Population-based and experimental validation of interactome-predicted drugs. (**a-c**) Drug-outcome evaluation using the Northwestern Medicine Enterprise Data Warehouse (NMEDW) and Cleveland Clinic Foundation (CCF) COVID-19 databases. Odds ratio was used to evaluate the carvedilol effect to the positive laboratory test result of COVID-19. Patients were matched with propensity score using age, gender, race, and other comorbidities (Table 1) to reduce various confounding factors. (**d**) Experimental validation of the anti-SARS-CoV-2 activity of carvedilol showed an EC_50_ value of 4.1 μM and low cell toxicity. EC_50_, half maximal effective concentration; CC_50_, half maximal cytotoxic concentration; SI, selectivity index (SI = CC_50_/EC_50_).

To identify the drug-outcome relationships of these drugs, we used a state-of-the-art active user-design approach^49,90^ based on large-scale electronic health record (EHR) data. Using the Northwestern Medicine Enterprise Data Warehouse (NMEDW) COVID-19 dataset (481,526 total patients, 66,541 COVID-19 positive cases, Table 1), we found that both carvedilol (odds ratio [OR] = 0.8, 95% confidence interval [CI] 0.68–0.94, *P* = 0.008) and hydrochlorothiazide (OR = 0.62, 95% CI 0.47–0.82, *P* < 0.001) were associated with a significantly lowered risk of positive COVID-19 test after confounding adjustment (age, sex, race, and comorbidities) using a propensity score (PS) matching approach^49,90^ (Fig. 4a–b and Table S9). The effect of carvedilol was consistent for different race and sex subgroups (Fig. 4a and Table S9). To validate these observations, we used a second EHR database from the Cleveland Clinic COVID-19 registry as an external validation set (168,712 total individuals, 83,340 SARS-CoV-2 positive cases, **Table S10**). We found that carvedilol had a sufficient number of usage cases for the drug-outcome evaluation. By comparing individuals with and without carvedilol usages (PS-matched by age, sex, race, and/or comorbidities), we found that carvedilol usage was associated with a 17% (OR = 0.83, 95% CI 0.78–0.88, *P* < 0.001) significantly lowered risk of COVID-19 positive test (Fig. 4c). This protective effect was also consistent when we examined subgroups from the registry in terms of race and sex (Fig. 4c).

**Extended Figure 4.**
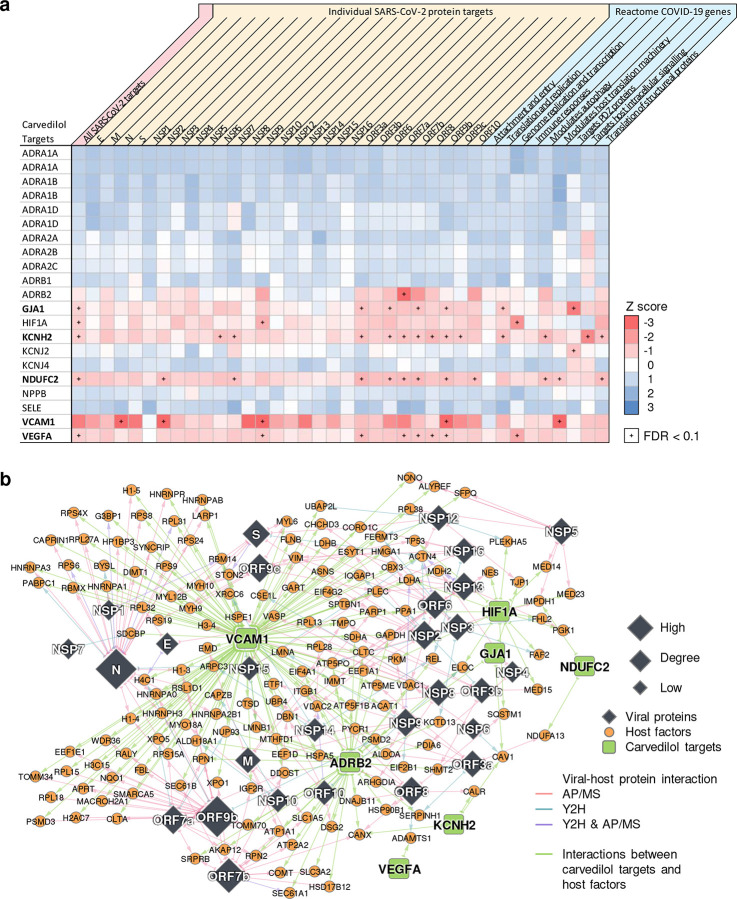
Carvedilol indirectly targets the SARS-CoV-2 host factors through protein-protein interactions with its targets. (**a**) Individual target-level network proximities to the SARS-CoV-2 gene sets (all host factors, host factors for each viral protein, and gene sets by different functions from Reactome). Network proximities were computed using the “shortest” method (See **Methods**). (**b**) Potential mechanisms-of-action of carvedilol by exploring the protein-protein interactions of its targets and the SARS-CoV-2 host factors.

We found that carvedilol not only showed favorable results in the EHR-based validation, but also has a promising antiviral profile from NCATS, showing high potencies for multiple desired activities (Fig. S5). The NCATS profile of carvedilol is comparable to that of remdesivir, whose profile was deemed highly desirable^91^. We then investigated carvedilol’s anti-SARS-CoV-2 activity experimentally. We treated A549-ACE2 cells with 0.3–20 μM of carvedilol for 2 hours followed by infection with SARS-CoV-2 at a multiplicity of infection (MOI) of 0.5 and incubation for 2 days. Carvedilol showed a low cell toxicity (Fig. 4d). Cells were subsequently fixed and immunostained to detect for S protein, which was used as a marker for infection. We found that carvedilol inhibited SARS-CoV-2 infection with an EC_50_ value of 4.1 μM (Fig. 4d), mechanistically supporting our SARS-CoV-2-human interactome-based prediction and EHR-based findings. Lastly, we conducted drug-target network analysis of carvedilol’s targets and SARS-CoV-2 host factors (Extended Fig. 4 and **Table S11**). We found that carvedilol could potentially affect the SARS-CoV-2 host factors (i.e., VCAM1 and KCNH2) through PPIs with its targets (**Extended Fig. 6**).

## Discussion

In this study, we leveraged high-throughput Y2H and quantitative TMT-AP/MS to generate the first binary and co-complex SARS-CoV-2-human protein-protein interactome network, expanding the known map produced solely by label-free AP/MS. This interactome validated 218 previously published SARS-CoV-2 host factors, and more importantly, revealed 361 novel ones. By comparing with previous interactomes, this interactome has higher overlaps among the interactomes and differentially expressed genes captured by bulk and single-cell RNA-seq of SARS-CoV-2 infection. The host factors identified in this interactome, particularly those altered in response to SARS-CoV-2 infection, present an invaluable opportunity for understanding the disease pathobiology of COVID-19 and prioritizing potential drug targets for treatment development.

Among the novel interacting partners for S protein, we identified several human proteins which may play important roles in SARS-CoV-2 infection. CORO1C^33^ and STON2^34^ are expressed on the cell membrane. CORO1C is highly expressed in lung (Table S5). STON2 is ubiquitously expressed and involved in endocytic machinery^34^. It is possible that SARS-CoV-2 can enter host cells through binding of S protein not only to ACE2, NRP1^92,93^ and BSG^94^, but also other (unknown) factors such as CORO1C and STON2. We also noticed two proteins, EPPK1^95^ and SPECC1L^96^, that both express on the cell junctions. It has been suggested that SARS-CoV-2 could spread through cell-to-cell transmission^97^. These cell junction proteins that can be targeted by SARS-CoV-2 S protein may facilitate its cell-to-cell transmission.

We identified a previously uncharacterized human transcription factor, ZNF579, that interacts with SARS-CoV-2 accessory protein ORF3a, and report that this interaction leads to the de-repression of *HSPA6*. Notably, HSPA6 is significantly upregulated after SARS-CoV-2 infection in cell culture models^40^, indicating that the disruption of ZNF579 by ORF3a may be relevant in the context of infection. HSPA6 is a HSP70 family molecular chaperone, which are known to be involved in the entry, replication, assembly, and release of various viral pathogens^98^. We speculate that SARS-CoV-2 has evolved this activity to ensure sufficient levels of molecular chaperones are available to assist with the production of viral proteins in cells.

**Extended Figure 5.**
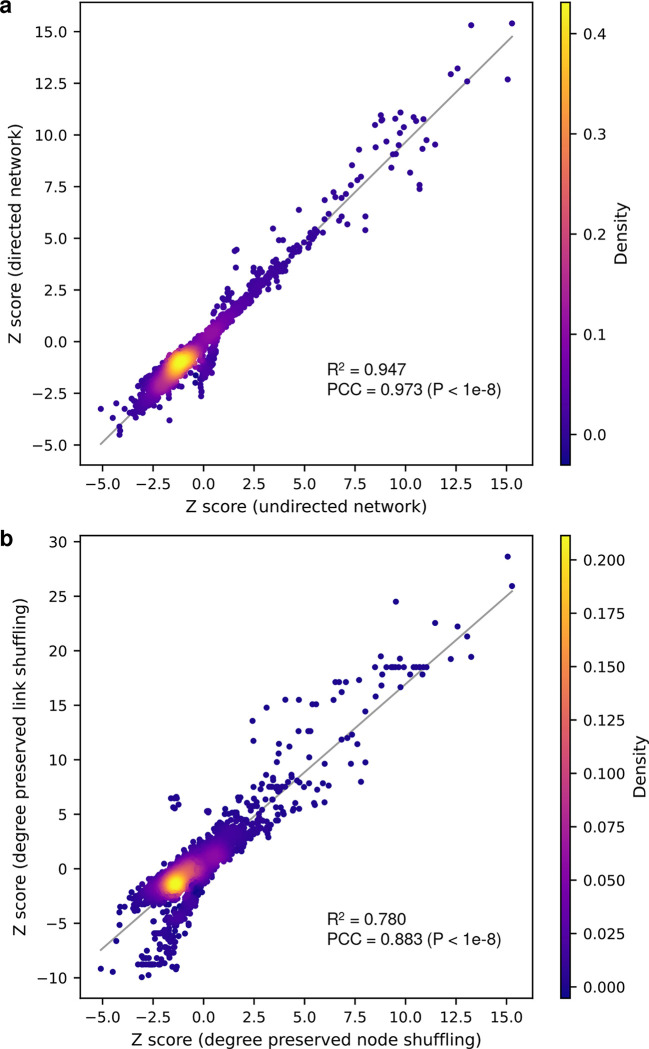
Comparison of the drug screening results using different variations of the network proximity-based screening methods. (**a**) Network proximity-based drug screening using directed human protein-protein interactome vs. undirected human protein-protein interactome. (**b**) Network proximity-based drug screening using degree preserved edge shuffling vs. degree preserved node shuffling. PCC, Pearson correlation coefficient.

Next, using this newly discovered SARS-CoV-2-human protein-protein interactome, we performed drug repurposing and identified a list of top 23 candidate drugs with known clinical trial evidence. In this study, we used undirected human protein interactome network and degree preserved node shuffling technique. We also tested different variations of the proximity analysis and found that using directed human protein interactome network and using degree preserved link shuffling resulted in overall highly consistent Z-scores compared to the original results in this study (Extended Fig. 5). We found that although some of these drugs can directly target the host factors, most of them indirectly affect the host factors through PPIs with their targets (Fig. 3b). For example, our predicted drug candidates are validated by well-established NCATS assays (Fig. S5). In addition, among the drugs which did not have NCATS assay results, alprazolam (Iranian Registry of Clinical Trials: IRCT20211015052773N1), L-Citrulline (ClinicalTrials.gov: NCT04404426 and NCT04570384), nadroparin (European Union Clinical Trials Register: EUCTR2020-001709-21-FR, EUCTR2020-001739-28-BE, EUCTR2020-005884-29-IT), vortioxetine (NCT05047952), and myrrh (Clinical Trials Registry - India: CTRI/2020/07/026669, CTRI/2020/12/029575, CTRI/2021/01/030825, Australian New Zealand Clinical Trials Registry: ACTRN12622000215729) are in clinical trials for COVID-19. Nevertheless, future experimental and clinical studies for novel predicted drug candidates are highly warranted.

Further, we have identified carvedilol and hydrochlorothiazide as potential host-targeting treatments for COVID-19 supported by multiple lines of evidence (strong network proximities to SARS-CoV-2 host factors, significantly reduced SARS-CoV-2 positive test risks in patients using these drugs based on large-scale EHR data, and experimental validation of anti-SARS-CoV-2 activity). As drug repurposing focuses on drugs that are already in existing patient databases, we are able to test hypotheses using EHR data as we^90,99^ and other teams^100,101^ demonstrated. The unique strengths of EHRs include their provision of large patient populations useful for detecting small differences and the availability of a large number of patient factors recorded without risk of recall bias, allowing for high-dimensional covariate adjustment to minimize confounding^49,100,102^. Our findings are consistent with previous reports that hydrochlorothiazide^100^ and carvedilol^101^ have potential beneficial effects for COVID-19 patients. Another beta-blocker metoprolol (Z = −2.327, FDR = 0.003) was also among the top 189 drugs (Table S7), which has been tested in a small clinical trial with positive effects^103^. These results confirm that the unique integration of SARS-CoV-2-human interactome findings and patient analysis approaches using two large-scale EHR databases from two independent health care systems, along with *in vitro* anti-viral observations, offer a powerful strategy for COVID-19 therapeutic discovery. This kind of systems biology strategy can be applied to future pandemics as well.

To understand the potential mechanisms of carvedilol’s anti-SARS-CoV-2 activity, we examined the carvedilol’s mechanism-of-action impacted by SARS-CoV-2 host factors using network analysis (Extended Fig. 4 and **Table S11**). Among the 579 unique host factors, 237 (41%) have PPIs with carvedilol targets. A large portion of the human proteins in the enriched pathways (protein translation [26/37, 70%], mRNA splicing [14/21, 67%], glucose metabolism [9/15, 60%], and neutrophil mediated immunity [14/27, 52%]) have PPIs with carvedilol targets, suggesting a potential mechanism-of-action in which carvedilol inhibits SARS-CoV-2 replication through multiple important pathways such as protein translation and mRNA splicing. We found several carvedilol targets that exhibited closer network distance to the virus host factors and COVID-19 pathways, such as GJA1, KCNH2, NDUFC2, VCAM1, and VEGFA (Extended Fig. 4). For example, VCAM1 plays important roles and has elevated levels in COVID-19^104,105^, and carvedilol can inhibit expression of VCAM1^106^. These results offer hypotheses that can be tested for the anti-SARS-CoV-2 effect of carvedilol. Yet, future experimental validation to decipher the anti-SARS-CoV-2 mechanism-of-action of carvedilol is highly warranted as well^106^.

We acknowledge several limitations. The network-based SARS-CoV-2 treatment discovery may be affected by the incompleteness of the human protein-protein interactome and drug-target network. Therefore, we relied not only on the network discoveries, but also incorporated other types of evidence, such as EHR-based validation and experimental validation. Our EHR-based validation is retrospective and can only be applied to commonly used drugs due to data availability. Although we adjusted for several confounding factors, other unknown factors may still have effect on the results of EHR-based validation. Therefore, the drugs identified in this study must be validated using randomized clinical trials before they can be used in patients with COVID-19. Lastly, we focused on the SARS-CoV-2-human protein interactome in this study. Combining multiple data resources (such as CRISPR^107^, genome-wide association studies^108^, rare variants^109^, synthetic lethality-based genetics interactions^110^, and metabolomics and proteomics^111^) may help identify comprehensive knowledge of COVID-19 using various advanced computational (e.g., genome-scale metabolic modeling^112^) and multi-omics data integration approaches.

## Materials and Methods

### SARS-CoV-2 ORF clones

ORF3b (plasmid no. 141384; Addgene), NSP4 (plasmid no. 141369; Addgene), NSP12 (plasmid no. 141378; Addgene), NSP13 (plasmid no. 141379; Addgene), and NSP14 (plasmid no. 141380; Addgene) were a gift from Nevan Krogan, University of California, San Francisco. NSP6 (plasmid no. 149309; Addgene) and NSP16 (plasmid no. 141269; Addgene) were a gift from Fritz Roth, University of Toronto, which we cloned into our pHAGE-CMV-GAW-3xMyc-IRES-PURO construct using Gateway. E, M, N, NSP1, NSP2, NSP3, NSP5, NSP7, NSP8, NSP9, NSP10, NSP15, ORF3a, ORF6, ORF7a, ORF7b, ORF8, ORF9b, ORF9c, ORF10, and S, cloned into pCAG-FLAG and pcDNA6B-FLAG constructs, were a gift from Pei-Hui Wang, Shandong University. All SARS-CoV-2 ORFs were codon-optimized and expressed in either pLVX-EF1alpha-eGFP-2xStrep-IRES-Puro (plasmid no. 141395; Addgene), pHAGE-CMV-GAW-3xMyc-IRES-PURO, pCAG-FLAG, or pcDNA6B-FLAG mammalian expression vectors.

### Y2H

Y2H screens were carried out as previously described^10,23–26^. In brief, viral ORFs were cloned into pDEST-AD and pDEST-DB vectors using Gateway LR to generate N-terminal ORF fusions. Similarly, human ORFeome 8.1^22^ was cloned into pDEST-AD and pDEST-DB vectors. All AD and DB expression clones were transformed into Y2H *Saccharomyces cerevisiae* strains *MATa* Y8800 and *MATα* Y8930 (genotype: leu2-3, 112 trp1-901 his3Δ200 ura3-52 gal4Δ gal80Δ GAL2::ADE2 GAL1::HIS3@LYS2 GAL7::lacZ@MET2 cyh2R), respectively. To screen out autoactivating DB-ORFs, all DB-ORF *MATα* Y8930 transformants were mated pairwise against empty pDEST-AD *MATa* Y8800 transformants and scored for growth on SC-Leu-Trp+3AT and SC-Leu-Trp-Ade plates, where DB-ORFs that triggered reporter activity were removed from further experiments. To increase screening throughput, 24 human ORF AD or DB clones were pooled into single human ORF AD or DB wells, respectively. Viral ORF AD and DB clones were then mated pairwise against pools of human ORF DB and AD clones, respectively. Mated transformants were incubated overnight at 30 °C before being plated onto SC-Leu-Trp to select for mated diploid yeast. After another overnight incubation at 30 °C, diploid yeast was plated onto SC-Leu-Trp-His+3AT and SC-Leu-Trp-Ade selection plates. After another overnight incubation at 30 °C, plates were replica-cleaned and incubated again for three days at 30 °C for final interaction calling.

### PLATE-seq

Each colony was picked 6 times into 96-well plates containing 15 μL of 2.5 mg/mL Zymolyase (catalog no. E1004; Zymo Research) and incubated for 45 min at 37 °C followed by 10 min at 95 °C to prepare yeast cell lysate used as PLATE-seq DNA template. PLATE-seq was carried out as previously described^26^. In brief, plasmid(s) from individual wells of 96-well plates were PCR amplified using a plasmid-specific forward primer and a reverse primer consisting of a well-position-specific barcode and TruSeq 3′ sequencing adapter. Amplicons derived from the same 96-well plate were pooled and purified using QIAquick PCR Purification Kit (catalog no. 28104, Qiagen). Each amplicon pool was subject to Tn5 tagmentation to fragment the amplicons and append adapters consisting of a plate-specific barcode and TruSeq 5′ sequencing adapter. Tagmented DNA was purified using QIAquick PCR Purification Kit (catalog no. 28104, Qiagen) and pooled across all 96-well plates. These pools were then subjected to low-cycle PCR both to extend the TruSeq end adapters with sequences compatible for binding to the Illumina flow cell and to enrich for only DNA fragments consisting of TruSeq adapter sequences on both ends of the plate specific and well-position-specific barcodes. PLATE-seq libraries were paired-end sequenced on an Illumina MiSeq.

### Affinity purification

Caco-2 (HTB-37; ATCC) cells were cultured in EMEM (catalog no. 30–2003; ATCC) with 15% FBS (catalog no. 30–2020; ATCC) at 37 °C with 5% CO_2_. All 28 SARS-CoV-2 ORFs were codon-optimized and cloned into mammalian expression vectors that contained Strep, Myc, or FLAG affinity tags. SARS-CoV-2 ORF plasmids and corresponding empty vectors were individually transfected in biological duplicates into Caco-2 cells using Lipofectamine 3000 Transfection Reagent (catalog no. L3000001; Invitrogen) following manufacturer’s instructions. Cells were harvested 72 hr post-transfection and lysed using RIPA lysis buffer (50 mM Tris-HCl [pH 7.5], 150 mM NaCl, 1% (v/v) Nonidet P 40 Substitute, 5 mM EDTA, phosphatase inhibitor (catalog no. 4906845001; Roche), and protease inhibitor cocktail (catalog no. 11873580001; Roche)). Samples were incubated for 30 min at 4 °C and then centrifuged at 13,000 ×g for 15 min at 4 °C. Supernatants were collected and incubate with either MagStrep “type3” XT beads (catalog no. 24090–002; IBA Lifesciences), Myc-Trap Agarose (catalog no. yta-10; ChromoTek) or Anti-FLAG M2 Affinity Gel (catalog no. A2220; Millipore) overnight at 4 °C. Strep-tagged samples were washed with 10x Buffer W (catalog no. 2-1003-100; IBA Lifesciences) three times at 4°C. Myc- and FLAG-tagged samples were washed with RIPA buffer. Strep-tagged samples were eluted using 10x Buffer BXT (catalog no. 2-1042-025; IBA Lifesciences). Myc- and FLAG-tagged samples were eluted using IP elution buffer (100 mM Tris-HCl [pH 7.5], 1% (v/v) SDS) and incubated for 15 min at 65 °C. Other primary antibodies used in this study include c-Myc Monoclonal Antibody (catalog no. 13–2500; Invitrogen) and Monoclonal Anti-FLAG M2 Antibody (catalog no. F3165; Sigma-Aldrich).

### Proteomic sample preparation

IP eluates were reduced using 200 mM TCEP for 1 hr at 55 °C. Samples were then alkylated using 375 mM iodoacetamide for 30 min at room temperature in the absence of light. Samples were digested using Trypsin Gold, Mass Spectrometry Grade (catalog no. V5280; Promega) at an enzyme-to-substrate ratio of 1:100 and incubated overnight with nutation at 37 °C. Peptide concentrations were measured using Pierce Quantitative Colorimetric Peptide Assay (catalog no. 23275; Thermo Scientific). Samples were normalized and resuspended using 1M Triethylammonium bicarbonate (TEAB) for TMT experiments (catalog no. 90114; Thermo Scientific). Samples were labeled using TMT10plex Isobaric Mass Tagging Kit (catalog no. 90113; Thermo Scientific) at a (w/w) label-to-peptide ratio of 10:1 for 1 hr at room temperature. Labeling reactions were quenched by the addition of 5% hydroxylamine and immediately pooled and dried using a SpeedVac. Labeled peptides were enriched and fractionated using Pierce High pH Reversed-Phase Peptide Fractionation Kit according to the manufacturer’s protocol (catalog no. 84868; Thermo Scientific).

### LC-MS/MS

Fractions were analyzed using an EASY-nLC 1200 System (catalog no. LC140; Thermo Scientific) equipped with an in-house 3 μm C18 resin- (Michrom BioResources) packed capillary column (75 μm × 25 cm) coupled to an Orbitrap Fusion Lumos Tribrid Mass Spectrometer (catalog no. IQLAAEGAAPFADBMBHQ; Thermo Scientific). The mobile phase and elution gradient used for peptide separation were as follows: 0.1% formic acid in water as buffer A and 0.1% formic acid in 80% acetonitrile as buffer B; 0–5 min, 5%−10% B; 5–65 min, 10–55% B; 66–67 min, 55%−95% B; 67–68 min, 2% B; 68–72 min, 95% B; 72–80 min, 5% B; with a flow rate set to 200 nL/min. MS1 precursors were detected at m/z = 375–1500 and resolution = 120,000. A CID-MS2-HCD-MS3 method was used for MS^n^ data acquisition. Precursor ions with charge of 2+ to 7+ were selected for MS2 analysis at resolution = 30,000, isolation width = 0.4 m/z, maximum injection time = 50 ms, and CID collision energy at 35%. 6 SPS precursors were selected for MS3 analysis and ions were fragmented using HCD collision energy at 65%. Spectra were recorded using Thermo Xcalibur Software Version 4.1 (catalog no. OPTON-30965; Thermo Scientific) and Tune application version 3.0 (Thermo Scientific). Raw data were searched using Proteome Discoverer Software 2.3 (Thermo Scientific) against an UniProtKB human database containing all SARS-COV-2 proteins. Search parameters specified precursor mass and fragment mass tolerance of 15 ppm. Peptide-spectrum matches (PSMs) were searched with SEQUEST HT and Percolator and filtered at FDR < 1%.

### Downstream proteomic analysis

We developed a novel pipeline using a customized linear model (inspired by MSstatsTMT^113^) to identify high-confidence viral-host interactions from TMT-AP/MS datasets. Briefly, PSMs filtered at 1% FDR were selected for quantification by (1) the number of reporter intensity values per fraction, (2) percent isolation interference, and (3) precursor intensity values to select for one instance of a peptide peak. If more than one PSM passed these criteria, then the average of the reporter ion intensities per channel of these PSMs were taken to represent the quantification of the peptide peak. The reporter intensity values of selected PSMs were log transformed, weighed with their respective precursor intensities, and averaged to obtain protein level quantification values. Our pipeline’s novelty lies in its ability to retain useful information separated across fractions at the PSM level while ensuring no violation of the assumption of independence, such that our linear fixed-effects model with conditions (e.g., sample vs. control), as a fixed effect, can be utilized. An improved *p*-value calculation was used through Empirical Bayes estimation of prior variance as implemented in R limma package^114^.

The fold change (FC) and *p*-values obtained from this linear model-based approach are used to generate volcano plots for each viral bait protein compared to control. A baseline cutoff was set at a FC of greater than 2 and FDR of less than 10%, on top of which a hyperbolic curve is optimized using the distribution of the log-transformed FCs of all identified proteins to identify high-confidence interactors. As a result, the actual cutoffs used for each AP/MS experiment are often significantly more stringent than the baseline values. A PSM cutoff, along with a peptide-coverage percent cutoff (i.e., the percentage of all possible trypsin digested peptides, accounting for up to two missed cleavages that can be found), based on the number of the viral protein’s PSMs and peptide-coverage percentage, is also implemented prior to the optimization of this hyperbolic curve.

### Co-immunoprecipitation

HEK 293T (CRL-3216; ATCC) cells were cultured in DMEM (catalog no. 30–2002; ATCC) supplemented with 10% FBS (catalog no. 30–2020; ATCC) and incubated at 37 °C with 5% CO_2_. Cells were seeded onto six-well plates and grown until reaching 70–80% confluency. SARS-CoV-2 N, ORF3a, ORF7b, histone H1.4 or N+histone H1.4, Sec61 or ORF7b+Sec61, STT3A or ORF7b+STT3A, and empty vector controls were transfected into cells by combining 2 μg of DNA with 10 μL of 1 mg/mL PEI (catalog no. 23966; Polysciences Inc.) and 150 μL Opti-MEM (catalog no. 31985062; Gibco). After 24 hr incubation, cells were gently washed three times with DPBS (1X) (catalog no. 14040117; Gibco), resuspended with 200 μL cell lysis buffer (10 mM Tris-HCl [pH 8.0], 137 mM NaCl, 1% (v/v) Triton X-100, 10% (v/v) glycerol, 2 mM EDTA, and protease inhibitor cocktail (catalog no. 11873580001; Roche)) and incubated on ice for 30 min. Extracts were then cleared by centrifugation at 16,000 ×g for 10 min at 4 °C. To perform co-immunoprecipitation (co-IP), 100 μL cell lysate was incubated with 5 μL Red Anti-FLAG M2 Affinity Gel (catalog no. F2426; Millipore) overnight at 4 °C under gentle rotation. Bound proteins were then washed three times with cell lysis buffer, eluted with 50 μl elution buffer (10 mM Tris-HCl [pH 8.0], 1% (v/v) SDS) and incubated for 10 min at 65 °C. Cell lysates and co-IP samples were then treated with 6X SDS protein loading buffer (1 M Tris-HCl [pH 6.8], 10% (v/v) SDS, 50% (v/v) glycerol, 0.03% (v/v) bromophenol blue, and 10% (v/v) β-mercaptoethanol), subjected to SDS-PAGE, and transferred onto PVDF membranes (catalog no. GE10600023; Amersham). For immunoblotting analysis, V5 Tag Monoclonal Antibody (catalog no. R960–25; Invitrogen), c-Myc Monoclonal Antibody (catalog no. 13–2500; Invitrogen), Monoclonal Anti-FLAG M2 Antibody (catalog no. F1804; Sigma-Aldrich), or ZNF579 Polyclonal Antibody (catalog no. A303–275A; Bethyl Laboratories) were used at 1:1,000 dilutions.

### qPCR

293T cells were cultured as above, and ORFa-FLAG or empty vector were introduced with Lipofectamine 2000 Transfection Reagent (catalog no. 11668030; Invitrogen) using manufacturer instructions. Transfection experiments were performed in duplicate. Media was replaced 6 hours after transfection, and RNA was harvested using TRIzol Reagent (catalog no. 15596018; Invitrogen). Reverse transcription was performed with the Maxima First Strand cDNA Synthesis Kit for RT-qPCR, with dsDNase (catalog no. K1671; Thermo Scientific). qPCR was performed on a LightCycler 480 System using LightCycler FastStart DNA Master SYBR Green I (catalog no. 03003230001; Roche Diagnostics). We used two primer sets for *HSPA6* (FWD1: CAAGGTGCGCGTATGCTAC, REV1: GCTCATTGATGATCCGCAACAC, FWD2: CATCGCCTATGGGCTGGAC, REV2: GGAGAGAACCGACACATCGAA), and performed 3 technical replicates of 3 concentrations of cDNA (1:10, 1:100, 1:1000) for each replicate, and then compared expression levels normalized to GAPDH using the double-delta Cp method.

### Interactome comparative analysis

We compared our SARS-CoV-2-human interactome to a collection of three previously reported interactomes^5–7^, and compared with ours in terms of the overlap (Fisher’s exact test) with the differentially expressed genes in SARS-CoV-2 from several SARS-CoV-2 RNA-seq/proteomics datasets. These datasets include: (1) a single-cell dataset that contains CD8, Epithelial (Epi) Ciliated, Epi-Secretory, Epi-Squamous, Macro, Mono, and NK cells from BALF^39^. We performed comparisons of virus^+^ vs. virus^−^ cells for each cell type; (2) bulk RNA-seq of human bronchial epithelial cells infected with SARS-CoV-2^40^ (GSE147507), denoted as SARS2-DEG; (3) proteomic dataset of human Caco-2 cells infected with SARS-CoV-2^41^, denoted as SARS2-DEP. (4) bulk RNA-seq of upper airway from COVID-19 patients vs. non-COVID-19 patients (GSE156063)^42^, denoted as DE-NS; (5) bulk RNA-seq of peripheral blood mononuclear cell (PBMC) isolated from COVID-19 patients vs. non-COVID-19 patients (GSE157103)^43^, denoted as DE-PBMC. For differential expression analysis, a cutoff of |log_2_FC| > 0.5 and FDR < 0.05 was considered significant. We calculated the Jaccard index (J) and overlap coefficient (C)^115^ for two gene sets *A* and *B* as below:

(1) $$J=\frac{\left|A\cap B\right|}{\left|A\cup B\right|}$$

(2) $$C=\frac{\left|A\cap B\right|}{\text{min}(\left|A\right|,\left|B\right|)}$$

### Functional enrichment analysis

Functional enrichment of our SARS-CoV-2 host factors were analyzed using Enrichr^116^ against the Kyoto Encyclopedia of Genes and Genomes (KEGG) and Gene Ontology (GO) biological process data sets. Pathways and GO terms with FDR < 0.05 were considered significantly enriched. GO terms were summarized using Revigo^117^.

### Selective pressure and evolutionary rates

The nonsynonymous and synonymous substitution rate ratio (*dN/dS* ratio)^118^ and the evolutionary rate ratio^119^ of our SARS-CoV-2 host factors were evaluated as described in a previous study^120^. For *dN/dS* ratio, *dN/dS*<1 was considered purifying selection; *dN/dS*=1 was considered neutral evolution; and *dN/dS*>1 was considered positive Darwinian selection. The evolutionary rate ratio >1 was regarded as a fast rate and <1 as a slow rate^119^.

### Tissue gene expression specificity

We evaluated the gene expression specificity of the SARS-CoV-2 host factors in 33 tissues using the RNA-Seq data from GTEx V8 (https://www.gtexportal.org/home/)^51^. The expression specificity of gene *i* in tissue *t* was defined as

(3) $$z_{it}=\frac{E_{it}-E_{i}}{\sigma_{i}}$$

where *E*_*i*_ was the mean and *σ*_*i*_ was the standard deviation of gene *i*’s expression across all considered tissues, and *E*_*it*_ was the mean expression of gene *i* in tissue *t*.

### Construction of human protein-protein interactome and drug-target network

The human protein-protein interactome and the drug-target network were used to screen for drugs against the SARS-CoV-2 host factors. The human protein-protein interactome, composed of 17,706 protein nodes and 351,444 unique PPI edges was constructed in our previous studies^48,49,121,122^. Briefly, several types of high-quality PPI evidence gathered from public databases and datasets were considered, including: binary PPIs identified by high-throughput yeast two-hybrid in three datasets^49,123,124^; low- or high-throughput experimentally discovered kinase-substrate interactions from KinomeNetworkX^125^, PhosphoNetworks^126^, Human Protein Resource Database (HPRD)^127^, DbPTM 3.0^128^, Phospho.ELM^129^, and PhosphositePlus^130^; signaling networks identified using low-throughput experiments in SignaLink2.0^131^; protein complexes revealed by robust affinity purification-mass spectrometry in BioPlex V2.016^132^; and curated PPIs from Instruct^133^, IntAct^134^, BioGRID^135^, MINT^136^, PINA^137^, and InnateDB^138^ that were identified by yeast two-hybrid studies, affinity purification-mass spectrometry, protein three-dimensional structures, or low-throughput experiments. For comparison, we also built a directed version of the human protein interactome using the PPI direction information (including kinase-substrate and signaling networks) from PhosphositePlus^130^ and SignaLink2.0^131^.

The drug-target network was constructed using several data sources as described in our recent studies^48,49,121^: DrugBank database (v4.3)^139^, BindingDB^140^, ChEMBL (v20)^141^, Therapeutic Target Database (TTD)^142^, PharmGKB database^143^, and IUPHAR/BPS Guide to PHARMACOLOGY^144^. Binding affinities K_i_, K_d_, IC_50_ or EC_50_ ≤ 10 μM were used as cutoff for the drug-target interactions. All networks were visualized using Cytoscape 3.8.0^145^. Clinical trial information was retrieved from the International Clinical Trials Registry Platform (ICTRP, assessed in May 2022).

### Network proximity-based drug and drug combination screening

The “closest” network proximity measure was used to screen for 2,938 FDA approved or investigational drugs. The “closest” distance *d_AB_* for two gene/protein sets *A* (e.g., drug targets) and *B* (e.g., SARS-CoV-2 host factors) was calculated as:

(4) $$\langle d_{AB}\rangle =\frac{1}{∥A∥+∥B∥}\left(\sum_{a\in A}{\text{min}}_{b\in B}d(a,b)+\sum_{b\in B}{\text{min}}_{a\in A}d(a,b)\right)$$

where *d*(*a*,*b*) is the shortest path length of *a* and *b* in the human protein-protein interactome. Network proximity *d*_*AB*_ was further normalized to obtain a Z score using a permutation test with randomly selected proteins from the interactome with similar degree distributions to *A* and *B* (degree preserved node shuffling). Permutation tests were repeated 1,000 times. We prioritized drugs by Z < −2 and FDR < 0.05. For comparison, we also conducted degree preserved link shuffling using the double edge swap method to swap the links 10 × the size of the human protein interactome times. For individual drug target level network proximity to the disease modules, we used the “shortest” measure that measures the average shortest distances of a target to the disease proteins (host factors):

(5) $$\langle d_{AB}\rangle =\frac{\sum_{a\in A,b\in B}d(a,b)}{∥A∥\times ∥B∥}$$

The antiviral profiles of the prioritized drugs were retrieved from NCATS (https://opendata.ncats.nih.gov/covid19/assays). NCATS contains experimental high-throughput screening results for drugs from a series of screenings (some accompanied by counterscreens) to evaluate their anti-SARS-CoV-2 potential. We included the following screening results: SARS-CoV-2 cytopathic effect (CPE) and its counterscreen SARS-CoV-2 cytopathic effect (host tox Counter) / Cytotoxicity; human fibroblast toxicity (hCYTOX); spike-ACE2 protein-protein interaction (AlphaLISA) and its counterscreen spike-ACE2 protein-protein interaction (TruHit Counter); ACE2 enzymatic activity; SARS-CoV pseudotyped particle entry (CoV-PPE) and its counterscreen SARS-CoV pseudotyped particle entry counter screen (CoV-PPE_cs); MERS-CoV pseudotyped particle entry (MERS-PPE) and its counterscreen MERS-CoV pseudotyped particle entry counter screen (MERS-PPE_cs); and 3CL enzymatic activity. Based on the NCATS SARS-CoV-2 data, we further selected a list of top drugs from the network proximity-based prioritization that show ideal activities in at least two of these screenings.

### COVID-19 patient data observations

Two independent datasets revealed corroborating evidence for the drug carvedilol which was identified by our interactome prioritization framework. The first dataset (discovery dataset) was from the Northwestern Medicine Enterprise Data Warehouse (NMEDW). We first identified 512,198 patients who had SARS-CoV-2 reverse transcription-polymerase chain reaction (RT-PCR) test results recorded in NMEDW. Patients with a positive RT-PCR test were considered COVID-19 positive, where the earliest time of the test was recorded as the effective time. Patients that did not have any positive or presumptive positive RT-PCR tests and the latest PCR test was negative (excluding pending and undetermined results) were considered COVID-19 negative, where the latest time of the test was recorded as the effective time. By these metrics, 29,224 patients with pending or undetermined results were removed, yielding 482,974 patients of interest. We excluded patients without age or sex information yielding a cohort of 481,526 patients, 66,541 of which were COVID-19 positive. We then extracted the carvedilol (and other drugs) administration information for all patients in the final cohort. If a patient had a carvedilol administration record with an administration date in the 6-month time window leading to the effective RT-PCR result date and an administered dose > 0, the patient was considered carvedilol+. We also extracted comorbidity information of the cohort for propensity score (PS) matching, for which we used the Charlson Comorbidity Index (CCI). All comorbidities and corresponding patient numbers are listed in Table 1.

The second dataset (external validation dataset) was an institutional review board-approved COVID-19 registry dataset that included 168,712 individuals tested for SARS-CoV-2 infection (83,340 of which were positive cases) from March 8^th^ to May 26^th^, 2021, at the Cleveland Clinic in Ohio and Florida, United States (**Table S10**). Pooled oropharyngeal and nasopharyngeal swab specimens were used to test for SARS-CoV-2 by RT-PCR assay in the Cleveland Clinic Pathology and Laboratory Medicine Institute. All SARS-CoV-2 testing followed the guidelines established by the Centers for Disease Control and Prevention of United States. The dataset included baseline demographic information, medications, and COVID-19 test results. We used REDCap^146^ electronic data capture tools to extract the patient data from the electronic health records (EPIC Systems), and the data were manually checked by a professional team trained on uniform sources for the study variables. A carvedilol exposure group (carvedilol+) included patients that were actively taking carvedilol at the time of SARS-CoV-2 testing. Positive laboratory test results for COVID-19 were used as the primary outcome. PS was used to match age, sex, and race to reduce various confounding factors. Odds ratio was used to evaluate the carvedilol benefit to primary outcome. All analyses were conducted by matchit package in the R v4.1.0 platform.

### Anti-SARS-CoV-2 activity assay for carvedilol

A549 (CCL-185; ATCC) cells exogenously expressing angiotensin-converting enzyme 2 (ACE2) (A549-ACE2) were a gift from Benjamin R. Tenoever, Icahn School of Medicine at Mount Sinai. A549-ACE2 cells were cultured in DMEM (catalog no. 11965092; ThermoFisher) with 10% FBS (catalog no. 100–106; GeminiBio) and used for SARS-CoV-2 infection. SARS-CoV-2 virus (nCoV/Washington/1/2020) was provided by the Biocontainment Laboratory–University of Texas Medical Branch Galveston National Laboratory, Texas, United States. Vero E6 (CRL-1586; ATCC) cells were used to propagate and titer SARS-CoV-2. SARS-CoV-2 infections were performed under biosafety level 3 conditions at the Biocontainment Laboratory–University of Chicago Howard T. Ricketts Laboratory, Illinois, United States. A549-ACE2 cells cultured in DMEM with 2% FBS were treated with carvedilol for 2 hours at the indicated concentrations. Cells were infected with an MOI of 0.5 in media containing the appropriate concentration of drug. 48 hr post-infection, cells were fixed with 10% formalin (catalog no. 305–510; Fisherbrand), blocked, and probed with mouse anti-SARS-CoV-2-spike antibody (catalog no. GTX632604; GeneTex) diluted 1:1,000 for 4 hr, rinsed, and probed with anti-mouse-HRP (catalog no. MP7401; Vector Labratories) for 1 hr, washed, and then developed with DAB substrate (catalog no. 34065; ThermoScientifc) for 10 min. Spike positive cells (n>40) were quantified by light microscopy as blinded samples. A sigmoid fit was used to extract EC50 values using MATLAB.

To measure the effect of carvedilol on cell viability, cells were treated with various concentrations of carvedilol diluted in 2% DMEM for 48 hours. The drug solution was then removed, and cells were fixed with 10% formalin solution. The cells were stained with Crystal Violet 0.25% for 30 minutes. The plate was spun dried in a tabletop centrifuge and absorbance of each well was measured using a TECAN Infinite 200 Pro at 595 nm. The % survival was calculated relative to DMSO treated cells.

## Extended Data

## Supplementary Material

Supplementary Tables

1

## Figures and Tables

**Table 1.** Patient Characteristics of NMEDW dataset.

	All patients		SARS-CoV-2 positive patients	
	Carvedilol−	Carvedilol+	Carvedilol−	Carvedilol+
Total	478,536	2,990	66,289	252
Age	44.67 ± 21.74	67.37 ± 15.08	41.86 ± 21.09	63.52 ± 17.20
Sex, male	202,994 (42.4)	1,674 (56.0)	30,550 (46.1)	146 (57.9)
Race				
Black	41,858 (8.7)	725 (24.2)	6,136 (9.3)	73 (29.0)
White	343,549 (71.8)	1,927 (64.4)	46,493 (70.1)	143 (56.7)
Other	60,273 (12.6)	274 (9.2)	9,407 (14.2)	27 (10.7)
Comorbidity				
AIDS HIV	1,843 (0.4)	36 (1.2)	248 (0.4)	5 (2.0)
CD	32,555 (6.8)	1,336 (44.7)	3,498 (5.3)	103 (40.9)
CPD	87,868 (18.4)	1,249 (41.8)	12,078 (18.2)	123 (48.8)
CHF	26,973 (5.6)	2,042 (68.3)	3,331 (5.0)	172 (68.3)
Dementia	8,567 (1.8)	358 (12.0)	1,297 (2.0)	42 (16.7)
Diabetes w cc	20,670 (4.3)	1,359 (45.5)	3,031 (4.6)	139 (55.2)
Diabetes wo cc	54,322 (11.4)	1,629 (54.5)	8,301 (12.5)	150 (59.5)
HP	5,380 (1.1)	298 (10.0)	568 (0.9)	27 (10.7)
Malignancy	47,660 (10.0)	805 (26.9)	4,606 (6.9)	67 (26.6)
MST	23,690 (5.0)	413 (13.8)	2,385 (3.6)	36 (14.3)
MLD	29,730 (6.2)	583 (19.5)	3,711 (5.6)	53 (21.0)
MSLD	2,906 (0.6)	119 (4.0)	307 (0.5)	11 (4.4)
MI	7,913 (1.7)	630 (21.1)	1,017 (1.5)	54 (21.4)
PUD	11,785 (2.5)	309 (10.3)	1,289 (1.9)	32 (12.7)
PVD	23,925 (5.0)	1,158 (38.7)	2,626 (4.0)	99 (39.3)
RD	26,068 (5.4)	1,820 (60.9)	3,479 (5.2)	181 (71.8)
RhD	13,887 (2.9)	232 (7.8)	1,607 (2.4)	18 (7.1)

Age is shown as mean ± standard deviation. All other characteristics are shown as number of cases (percentage). P values were calculated by two-sided t test for age and Fisher’s exact test for other variables. AIDS HIV, acquired immunodeficiency syndrome and human immunodeficiency virus; CD, cerebrovascular disease; CPD, chronic pulmonary disease; CHF, congestive heart failure; Diabetes w/wo cc, diabetes with/without chronic complications; HP, hemiplegia or paraplegia; MST, metastatic solid tumor; MLD, mild liver disease; MSLD, moderate or severe liver disease; MI, myocardial infarction; PUD, peptic ulcer disease; PVD, peripheral vascular disease; RD, renal disease; RhD, rheumatic disease.

## Data Availability

We downloaded the GTEx v8 dataset from https://gtexportal.org/home/. The human protein-protein interactome and drug-target network are found in https://github.com/ChengF-Lab/COVID-19_Map. An interactive version of Fig. 1b can be found in https://github.com/ChengF-Lab/COVID-19_PPI. All other data can be found in the supplementary tables. We accessed ZNF579 ChIP-seq data from the ENCODE portal under accession ENCSR018MQH.
