# Supplementary material for: A comprehensive SARS-CoV-2-human protein-protein interactome network identifies pathobiology and host-targeting therapies for COVID-19
Source: Res Sq. 2022 Jun 7:rs.3.rs-1354127. Preprint. [Version 2] doi: 10.21203/rs.3.rs-1354127/v2 (PMC9176654; doi:10.21203/rs.3.rs-1354127/v2)
Supplement: 1 [file NIHPPRS1354127V2-supplement-1.pdf]

Figure S1

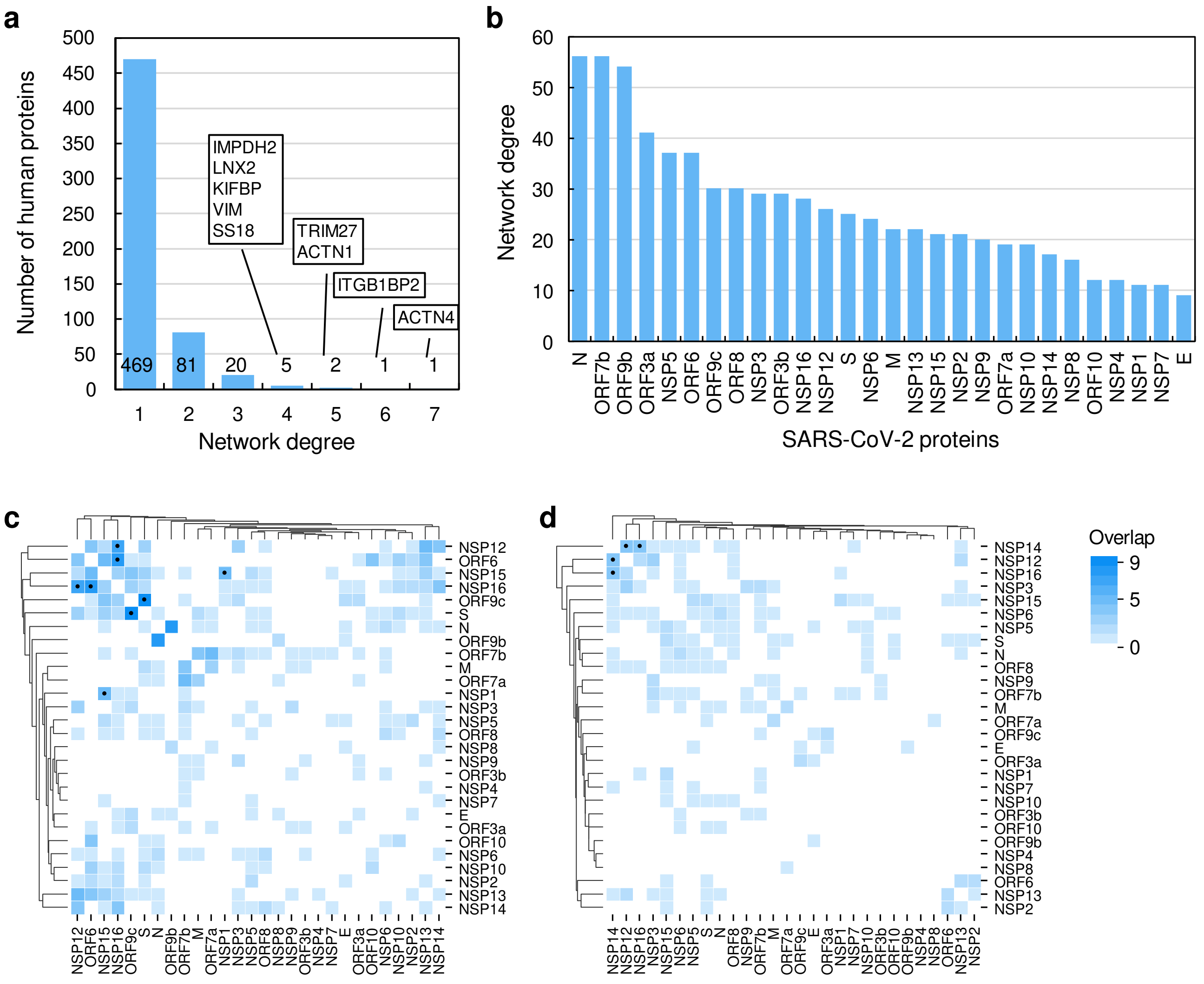

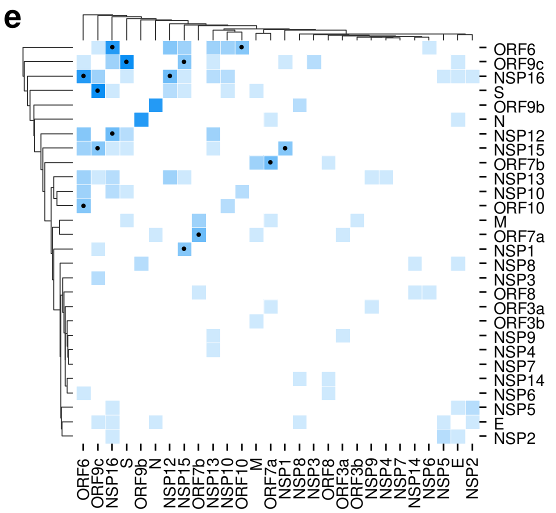

Figure S1. Summary statistics of the SARS-CoV-2-human protein-protein interactome. (**a**) Degree distribution of the human proteins. (**b**) Degree distribution of the viral proteins. (**c**) Number of overlapping targets of the viral proteins. Dot indicates FDR < 0.05 by Fisher's exact test. (**d**) Number of overlapping targets of the viral proteins revealed by Y2H. (**e**) Number of overlapping targets of the viral proteins revealed by AP/MS.

Figure S2

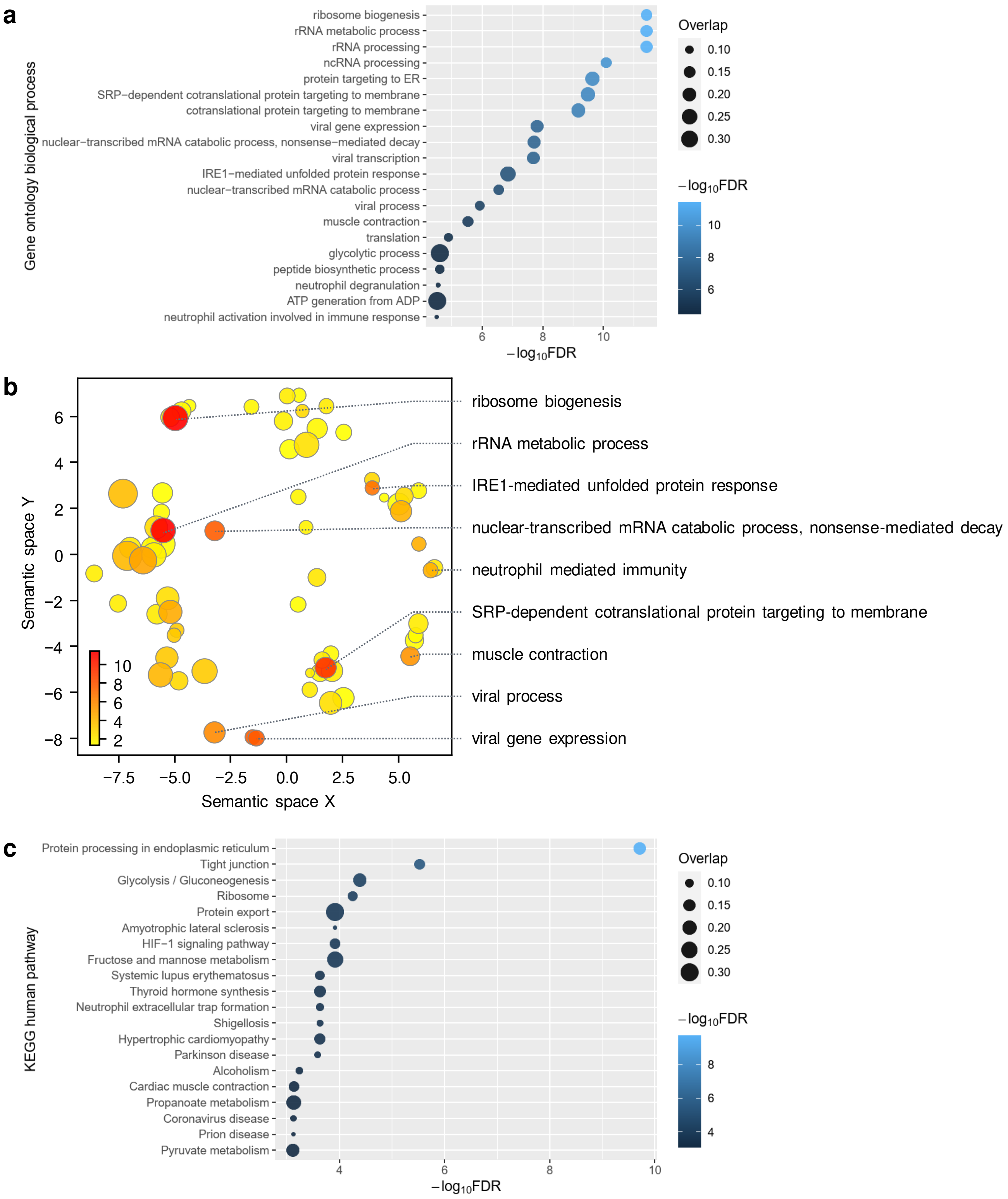

Figure S2. Pathway and gene ontology enrichment analyses for SARS-CoV-2 host factors. Enrichment analyses were performed with Enrichr. (**a**) Gene ontology (GO) biological process enrichment. (**b**) Summarization of the significant (FDR < 0.05) GO terms using Revigo. Color scale indicates -$\log_{10}$FDR. Dot size indicates $\log_{10}$(number of annotations for the terms in the EBI GOA database). (**c**) KEGG human pathway enrichment.

Figure S3

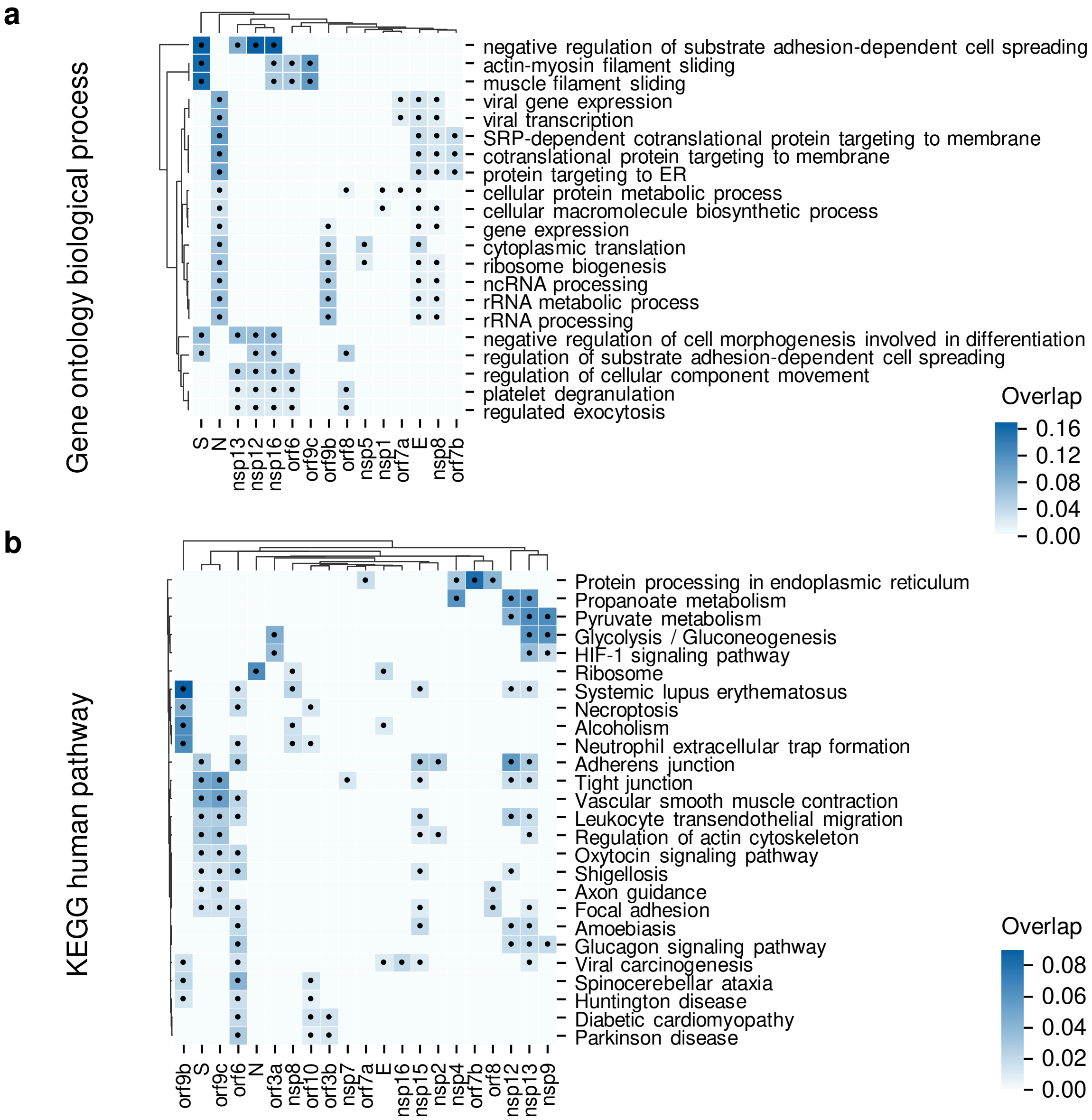

Figure S3. Pathway and gene ontology enrichment analyses for the host factors of individual SARS-CoV-2 viral proteins. Color shows % overlap of the host factors against the gene sets. Black dot indicates FDR < 0.05. Enrichment analyses were performed with Enrichr. Gene ontology (**a**) terms and pathways (**b**) that are significant in at least three viral proteins are shown.

Figure S4

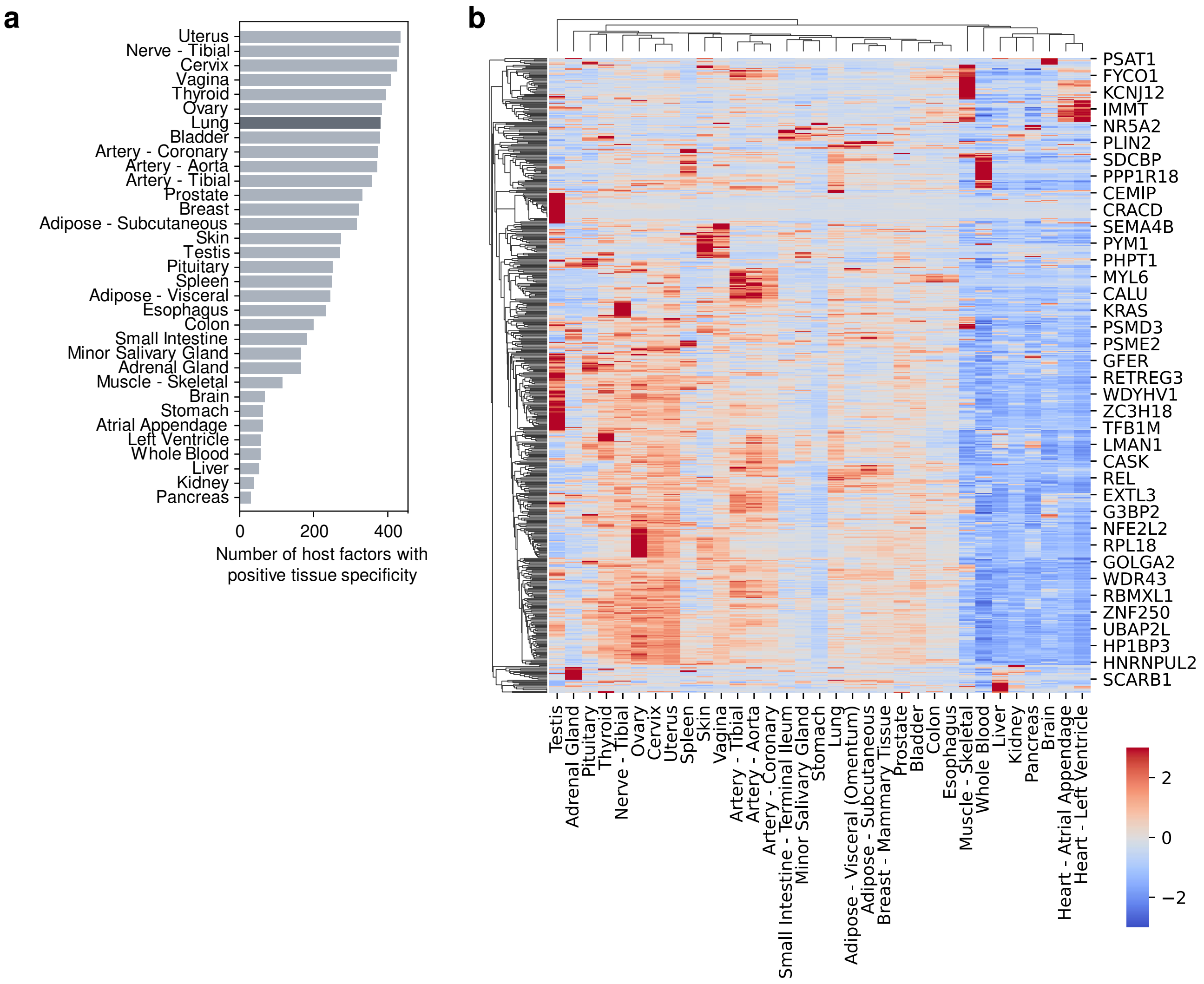

Figure S4. Overview of the expression of the host factors. (**a**) Ranking of the number of host factors with positive tissue specificity in each tissue. (**b**) Tissue-specificity of the host factors. RNA-Seq data in transcript per million were downloaded from GTEx V8 (https://www.gtexportal.org/home/). Data were z-score scaled for each gene across 33 tissues.

Figure S5

a. Carvedilol

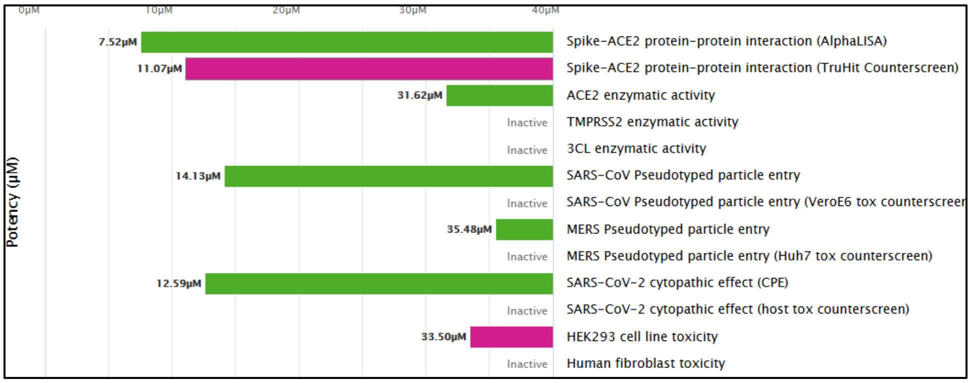

b. Apremilast

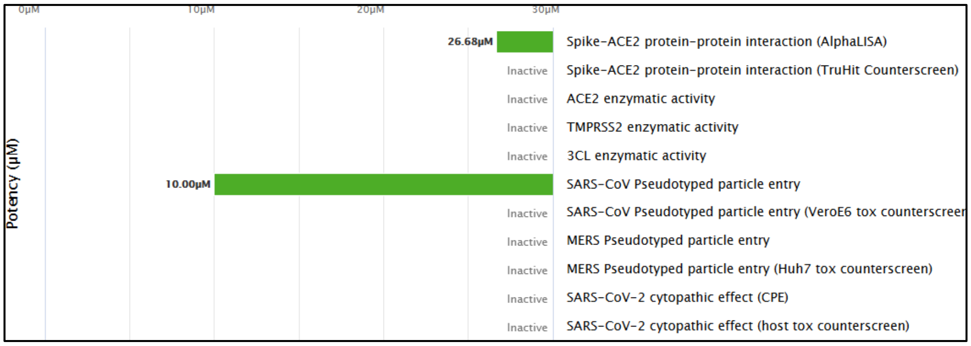

c. Mefenamic acid

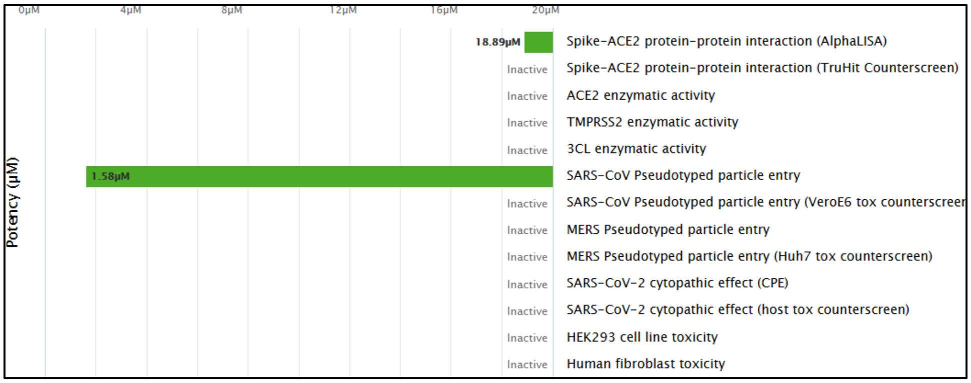

d. Balsalazide

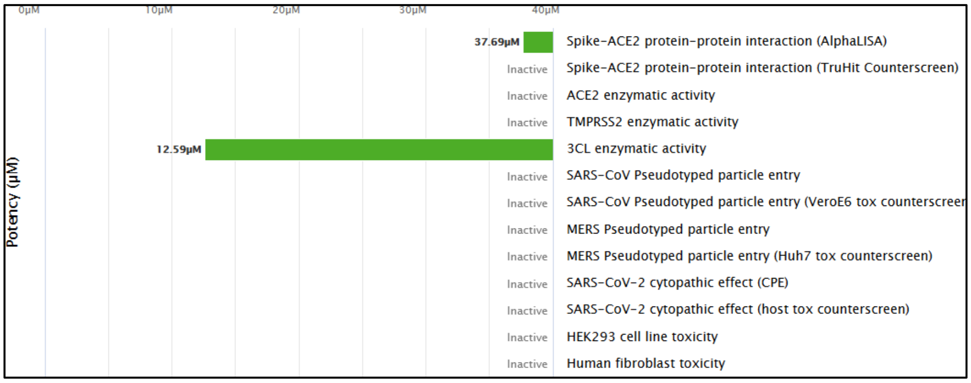

Figure S5, continued

e. Azithromycin

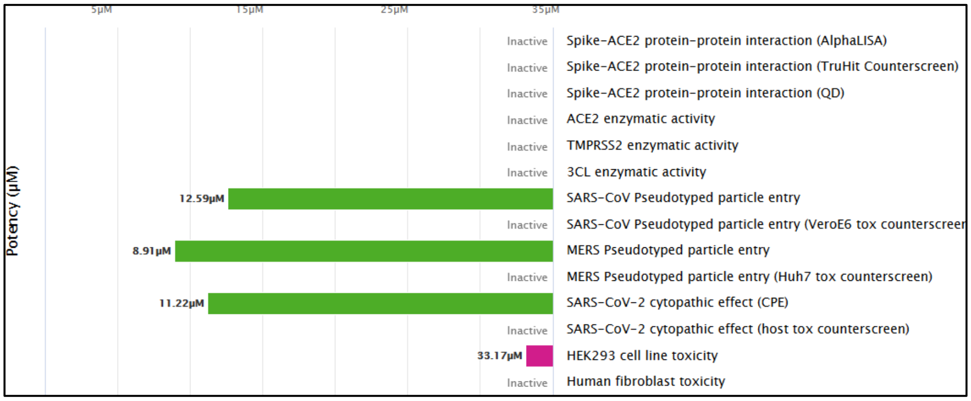

f. Toremifene

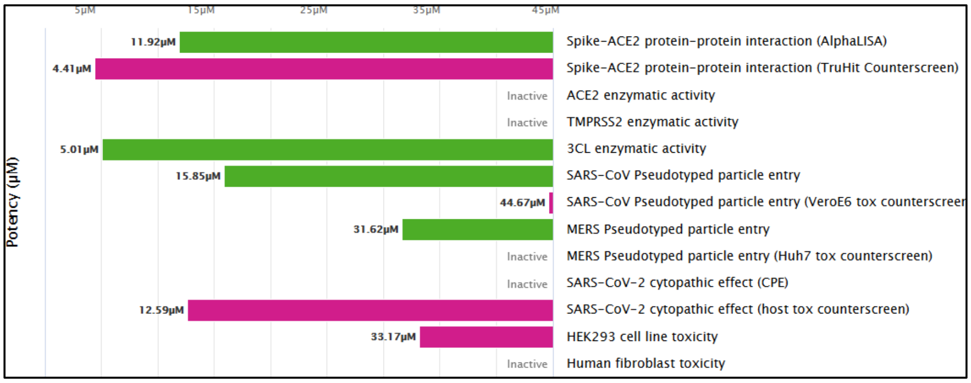

g. Hydrochlorothiazide

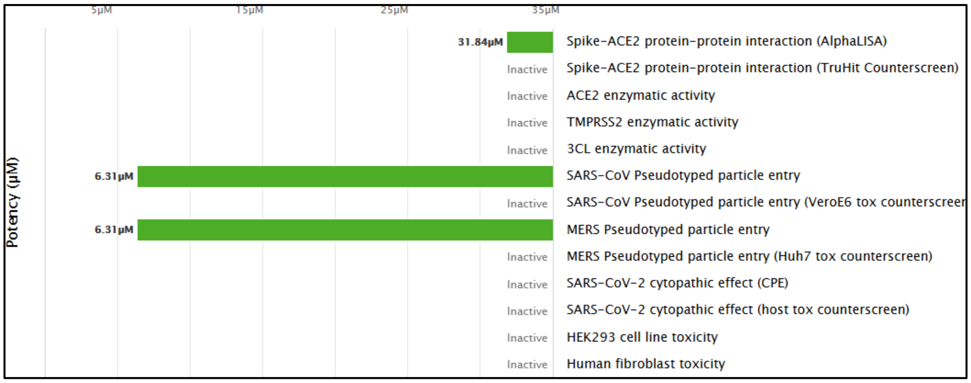

h. Nilvadipine

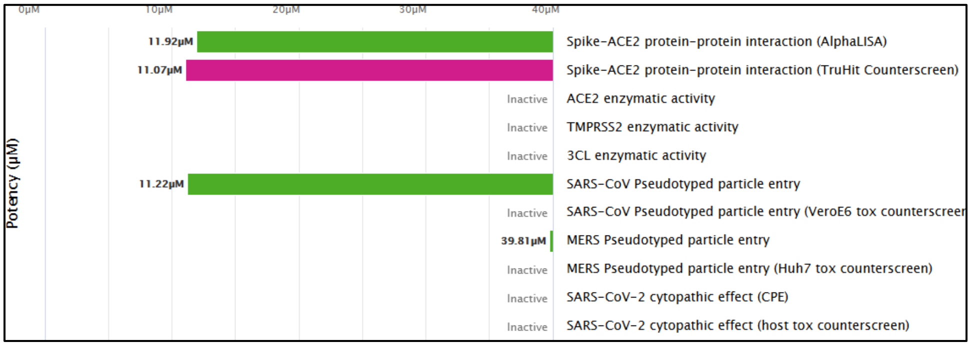

Figure S5, continued

i. Amodiaquin

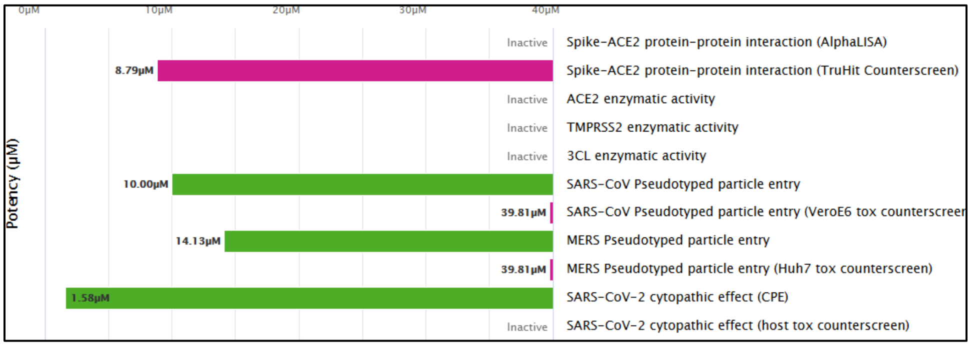

j. Tetracycline

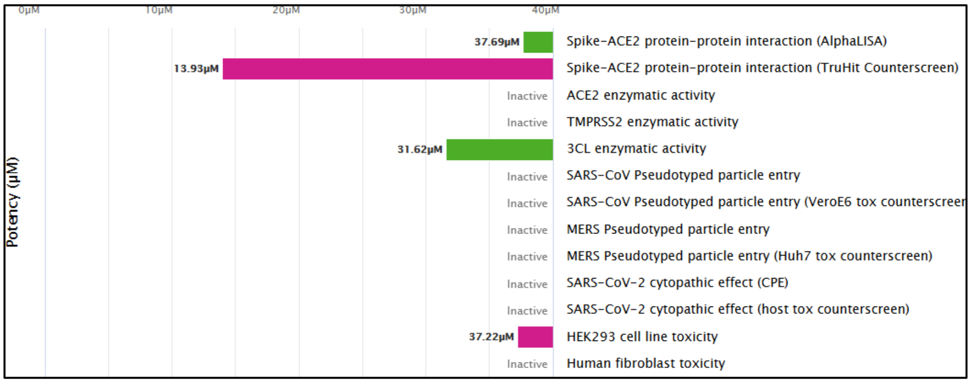

k. Xylometazoline

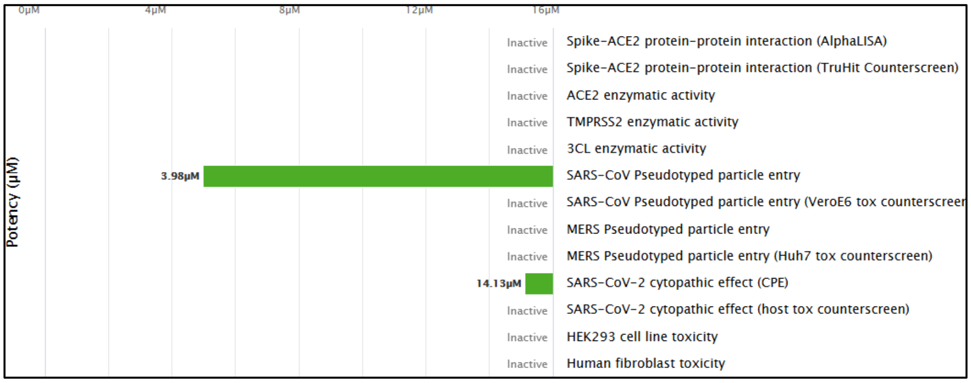

l. Decitabine

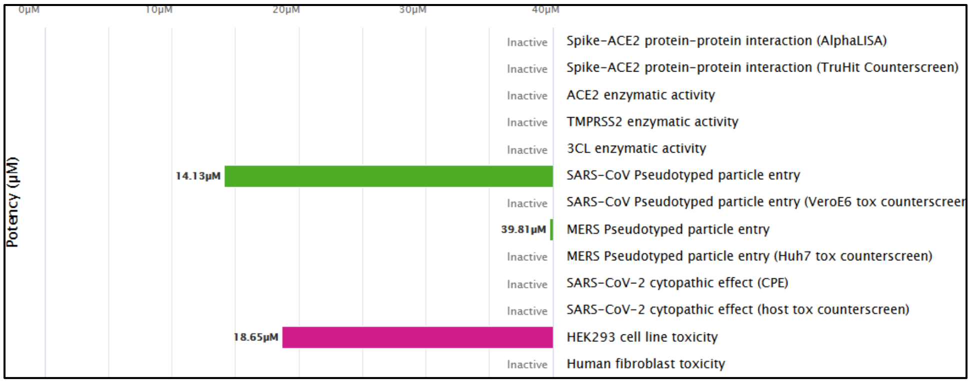

Figure S5, continued

m. Adefovir dipivoxil

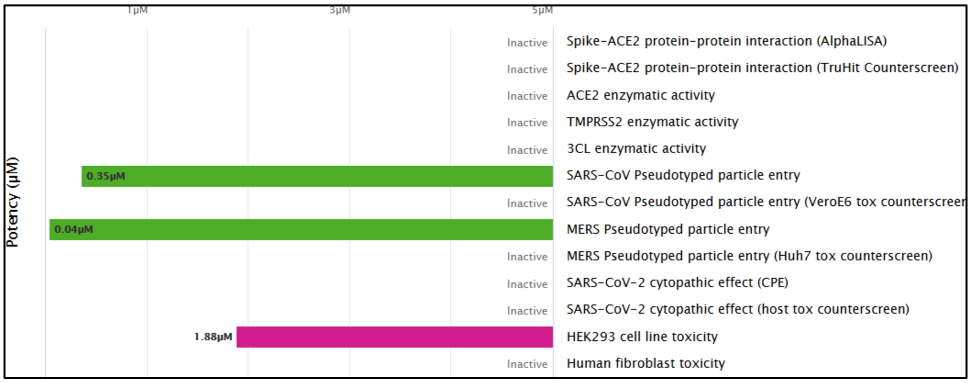

n. Venetoclax

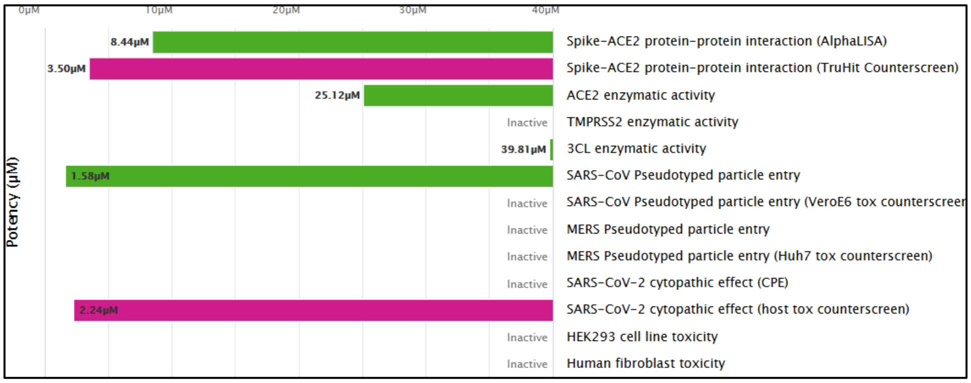

o. Calcipotriol

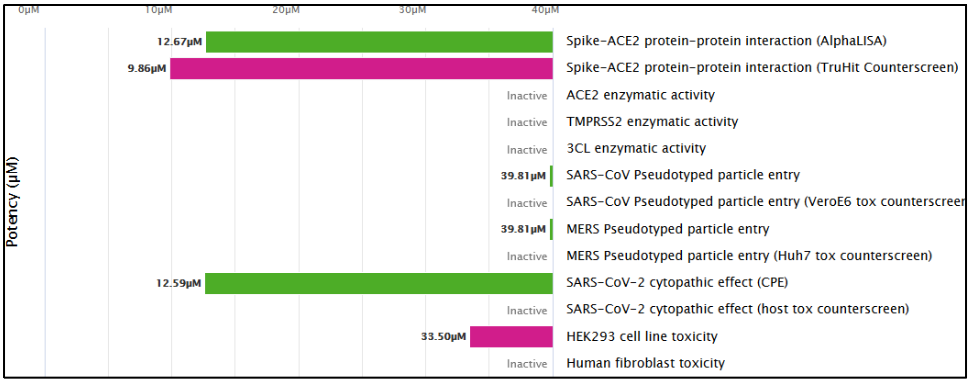

p. Amitriptyline

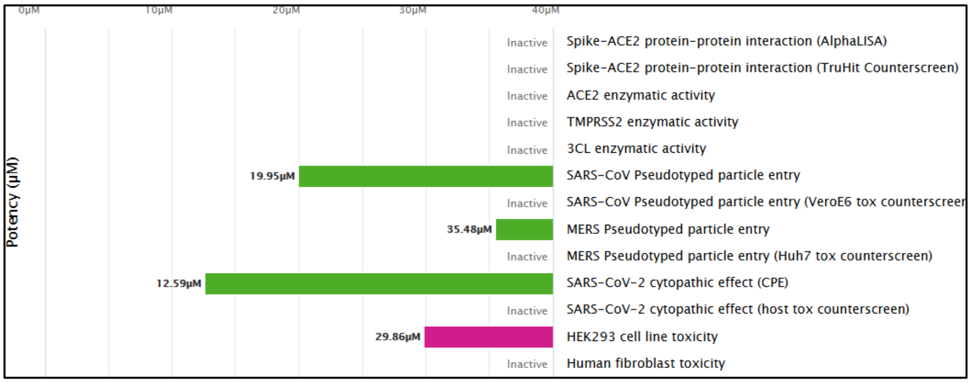

Figure S5, continued

q. Fenoprofen

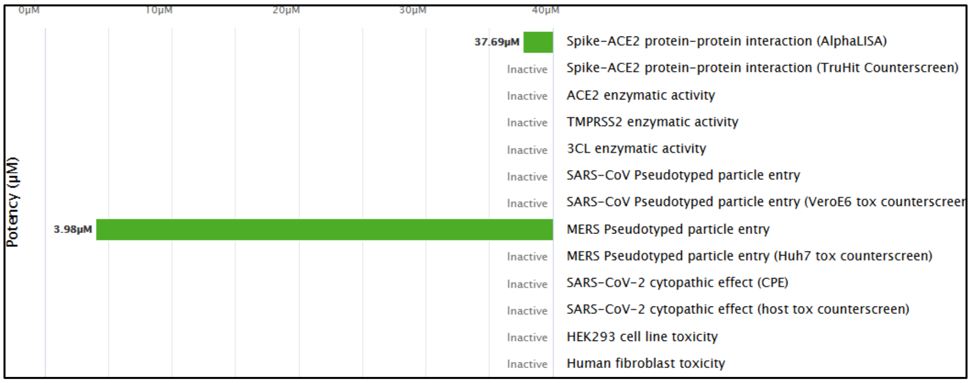

r. Tipranavir

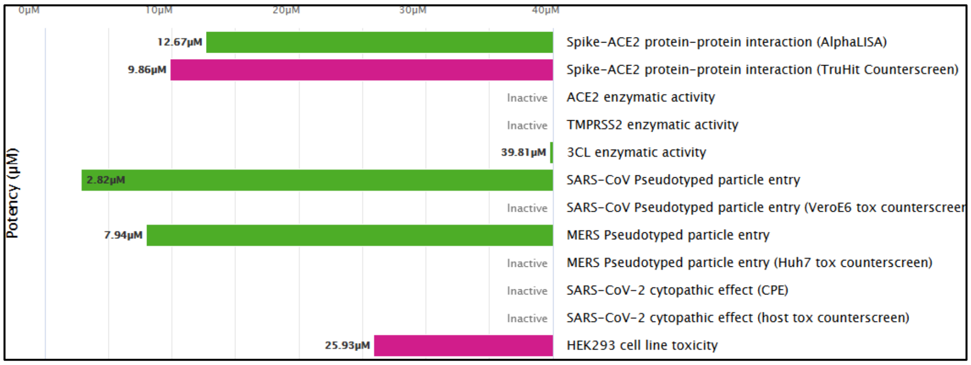

s. Probucol

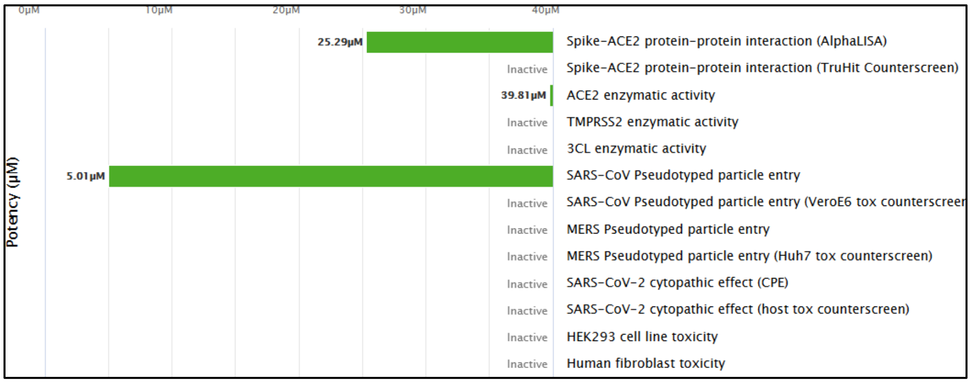

t. Brimonidine

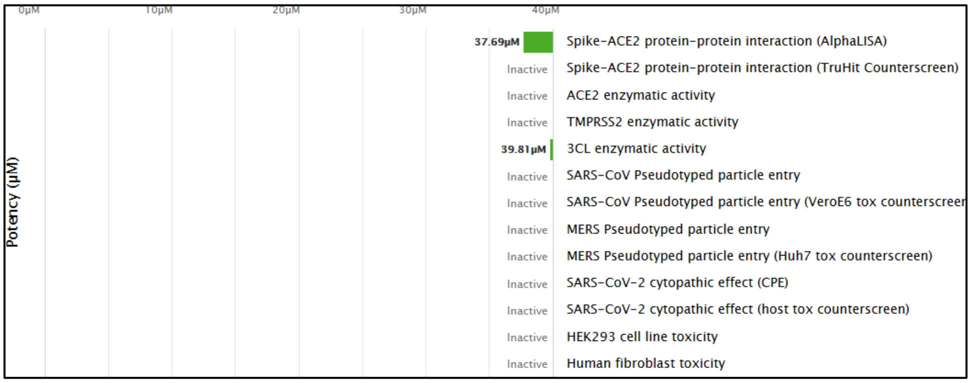

Figure S5, continued

u. Repaglinide

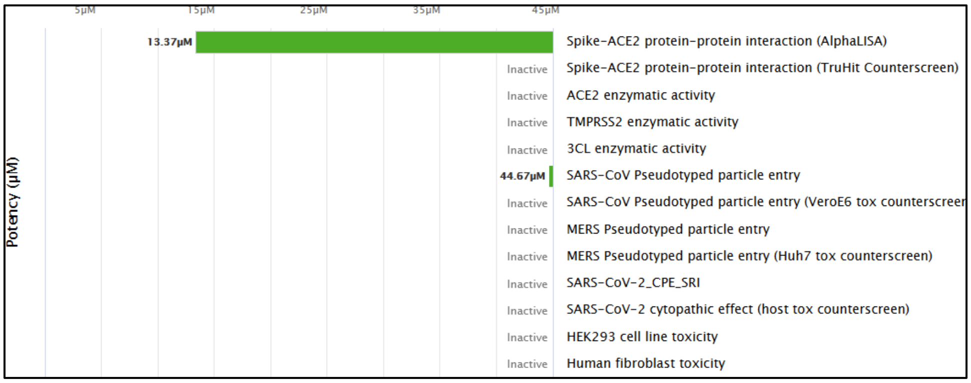

v. Dienestrol

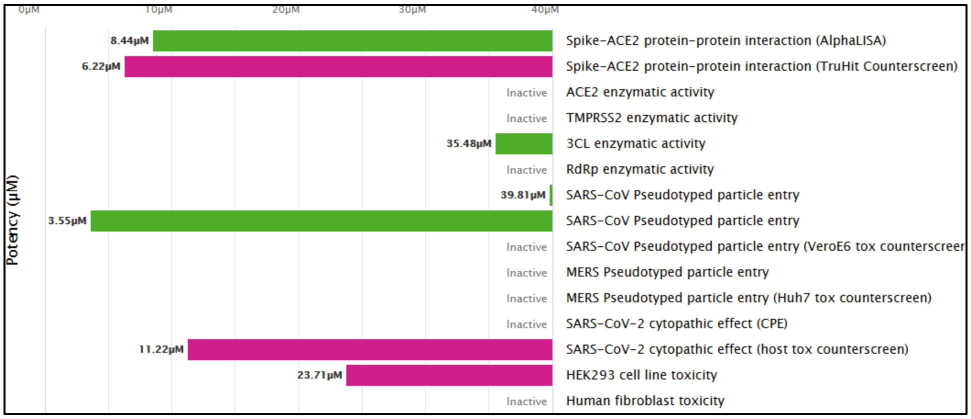

w. Lurasidone

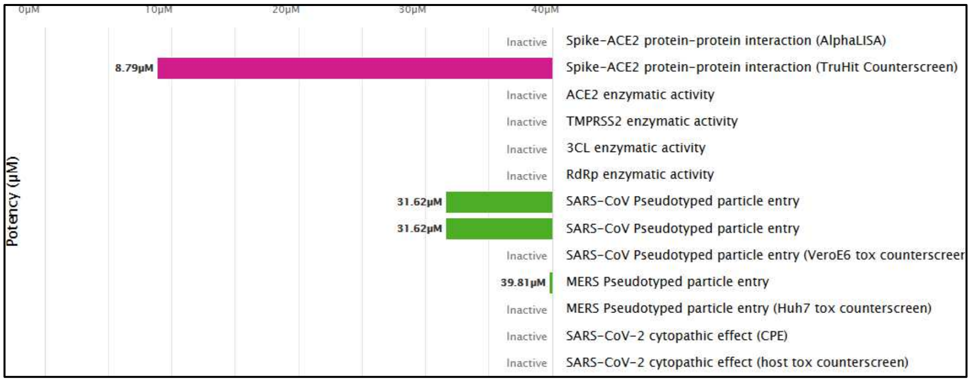

Figure S5. Anti-SARS-CoV-2 profiles for the top 23 drugs. Data were retrieved from NCATS (https://opendata.ncats.nih.gov/covid19/assays).

Figure S6

a. Norfloxacin

b. Isoconazole

c. Rucaparib

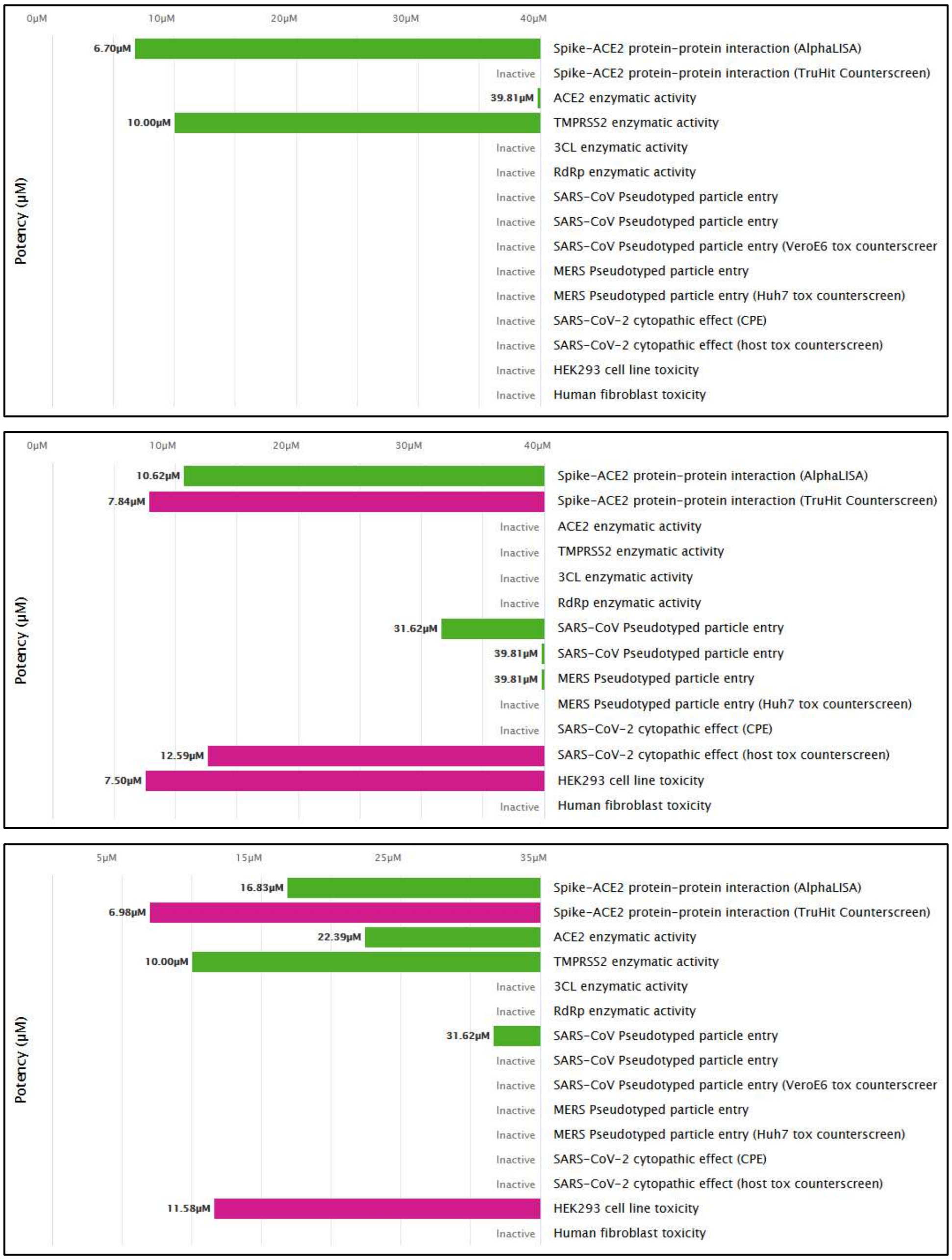

Figure S6. Anti-SARS-CoV-2 profiles for the three drugs identified by combining all four interactomes. Data were retrieved from NCATS (https://opendata.ncats.nih.gov/covid19/assays).
